# Association of retinopathy with risk of all-cause and specific-cause mortality in the National Health and Nutrition Examination Survey, 2005 to 2008

**DOI:** 10.3389/fpubh.2023.1200925

**Published:** 2023-08-23

**Authors:** Si-Yu Gui, Xin-Chen Wang, Jian-Chao Qiao, Si-Yu Lin, Qian-Qian Wang, Meng-Yue Zhang, Yue-Yang Xu, Zhi-Hao Huang, Li-Ming Tao, Cheng-Yang Hu, Fang-Biao Tao, Zheng-Xuan Jiang, Dong-Wei Liu

**Affiliations:** ^1^Department of Ophthalmology, The Second Affiliated Hospital of Anhui Medical University, Hefei, China; ^2^Department of Clinical Medicine, The Second School of Clinical Medicine, Anhui Medical University, Hefei, China; ^3^Department of Clinical Medicine, The First School of Clinical Medicine, Anhui Medical University, Hefei, China; ^4^Department of Humanistic Medicine, School of Humanistic Medicine, Anhui Medical University, Hefei, China; ^5^Department of Epidemiology and Biostatistics, School of Public Health, Anhui Medical University, Hefei, China; ^6^Department of Maternal, Child and Adolescent Health, School of Public Health, Anhui Medical University, Hefei, Anhui, China; ^7^Anhui Provincial Key Laboratory of Population Health and Aristogenics, Anhui Medical University, Hefei, Anhui, China

**Keywords:** retinopathy, all-cause mortality, specific-cause mortality, retinopathy status, risk factors

## Abstract

**Objective:**

This study aimed to elucidate the relationship between retinopathy status or severity and the all-cause and specific-cause mortality risk based on the updated National Health and Nutrition Examination Survey (NHANES) database and 2019 Public Access Link mortality file.

**Methods:**

In this prospective cohort study, a total of 6,797 participants aged over 40 years based on NHANES 2005–2008 were analyzed. The severity of retinopathy was classified into 4 grades-no retinopathy, mild non-proliferative retinopathy (NPR), moderate to severe NPR, and proliferative retinopathy (PR). Multiple covariate-adjusted Cox proportional hazards regression models and Fine and Gray competing risk regression models were used to assess the all-cause and cause-specific mortality risks, respectively. The propensity score matching (PSM) approach was also applied additionally to adequately balance between-group covariates to validate our findings.

**Results:**

A final total of 4,808 participants representing 18,282,772 United States (US) non-hospitalized participants were included for analysis, 50.27% were male (*n* = 2,417), 55.32% were non-hispanic white (*n* = 2,660), and mean [SE] age, 56.10 [0.40] years. After a median follow-up of 12.24 years (interquartile range, 11.16–13.49 years), 1,164 participants died of all-cause mortality, of which 941 (80.84%) died without retinopathy and 223 (19.16%) died with retinopathy at baseline. The presence of retinopathy was associated with increased all-cause mortality, cardiovascular disease (CVD), and diabetes mellitus (DM)-specific mortality, and the results remain consistent after PSM. Severity analysis showed that only mild NPR was associated with an increased all-cause mortality risk (hazard ratio (HR) = 2.01; 95% confidence interval (CI), 1.00–4.03), while increased CVD and DM-specific mortality risk were associated with all grades of retinopathy and were exponentially greater with increasing retinopathy severity, and the trend test was also significant (P for trend 0.004 and 0.04, respectively).

**Discussion:**

Our findings suggest that the diagnosis of retinopathy is an independent risk factor for all-cause mortality in people over 40 years old. Retinopathy grading is significantly associated with the survival risk of patients with CVD or DM, it can be a valuable predictor in the stratified management and risk warning of CVD or DM patients, as well as in the monitoring of systemic vasculopathy status.

## 1. Introduction

Retinopathy is a common ophthalmic disease that can be caused by retinal vasculopathy, blood circulation disorders, degeneration, and inflammation, etc. which is independently associated with a variety of diseases including diabetes mellitus (DM), coronary heart disease and hypertension. (1) Retinopathy is a major cause of visual impairment and blindness, with the typical clinical signs of microaneurysms, retinal hemorrhages and soft/hard exudates, more often in diabetic patients. The prevalence of diabetes among adults in the United States is estimated to have risen from 5.3% in 1976–1980 to 11.5% in 2011–2014, representing faster growth than the global rate over the same period. (2, 3) Diabetic retinopathy is a common microvascular complication of diabetes and a leading cause of blindness among working-age adults in the US. (4) A pooled analysis of 22,896 diabetic patients from 35 studies suggested an overall prevalence of 34.6% for any retinopathy. (5) A recent meta-analysis including 59 population-based studies estimated that 22.27% of diabetic patients worldwide have diabetic retinopathy (DR), which is expected to reach 160.5 million by 2045. (6) The disease burden of diabetes has increased in the United States in recent years, with early screening and ophthalmic examinations for diabetic retinopathy helpful in improving the prognosis of patients. In recent years, the disease burden of diabetic patients in the U.S. has been increasing, and fundus examination for diabetic retinopathy is one of the most important adjunctive measures in the screening, control, and treatment of diabetes. However, relevant studies in the U.S. have not yet utilized the early examination of retinopathy for the prediction of patient prognosis and mortality outcomes.

Despite the high prevalence of retinopathy and its serious impairment of vision, the underlying mechanisms of its development and progression are poorly understood. Epidemiological investigations and clinical trials have so far identified a number of risk factors such as blood pressure, (7, 8) oxidative stress, (9) lipids and obesity. (10) Retinopathy has been clearly associated with systemic vascular comorbidities (11–14) and as the only part of the body where blood vessels are directly visible, imaging screening of the retina may provide a convenient and early opportunity to assess the patient’s overall vascular disease or systemic disease burden. (15) In addition a number of studies have investigated the joint effect of retinopathy and systemic comorbidities on mortality. (16–18) However, given the different methods of assessing exposure and outcome as well as the different adjustments for confounders, the results of the relationship between the severity of retinopathy and overall and specific mortality are conflicting. First, most of the previous studies examined a smaller group of patients (usually with a sample size of less than 2,000) and considered only a dichotomous approach (retinopathy versus no retinopathy) without considering the severity of retinopathy and the risk of trend changes for overall and specific mortality. (15, 19, 20) Secondly, inconsistent results often resulted from inadequate adjustment for important confounders or the omission of some critical confounders, such as systemic cardiovascular disease (CVD) comorbidities and age-related ophthalmic comorbidities (cataract, age-related macular degeneration (AMD), glaucoma), social and economic level, depression and difficulty walking, etc. (16, 21) In addition, given that observational studies were unable to apply randomized grouping, most traditional study models have not adequately adjusted for the effects of important confounding factors between groups, leading to potential imbalances in confounding factors and systematic biases that remain difficult to eliminate. Furthermore, an even more important point is that most of the previous relevant studies may have overestimated the absolute risk of cause-specific mortality (e.g., CVD, DM), since they did not consider the competing risks of death. Given that a meta-analysis reported that the presence of retinopathy suggested a 2- to 4-fold increase in all-cause mortality risk independently of the influence of other potential risk factors. (22) Therefore, clarification of the precise impact of retinopathy and severity on the risk of future death, particularly from CVD and DM, is necessary to understand whether retinopathy can be used as a simple and effective predictor and to further develop early screening assessments as well as stratified risk management.

The National Health and Nutrition Examination Survey (NHANES), a population-based study providing a nationally representative sample of the U.S. noninstitutionalized population, has become a continuous survey study since 1999 and released every two years. Two previous published studies on related topics based on NHANES (after 1999) have been reported by Emily Frith et al. (23) and Zhuo-Ting Zhu et al. (24), but they were both based on limited median follow-up (4.58 and 8.33 years, respectively) and reported only on the relationship between all-cause mortality and retinopathy, lacked adjustment for important confounding factors (e.g., CVD and age-related ocular comorbidities) which may have led to overestimation of risks. Neither of them has carried out a standard grading of retinopathy severity. In conclusion, to our knowledge, there is still no comprehensive report on the relationship between specific causes of death and retinopathy (e.g., CVD, DM) by appropriate models, not to mention the analysis of retinopathy severity and trend difference tests, as well as the adjustment for important confounders. It is of greater clinical urgency and importance to clarify whether patients with specific diseases can benefit from fundus examination and retinopathy grading status. The aim of this study was to comprehensively elucidate the relationship between retinopathy and all-cause and cause-specific mortality by aggregating a range of demographic characteristics, health behaviors and characteristics, systemic comorbidities (e.g., CVD and ocular comorbidities) based on the updated NHANES follow-up mortality data, to explore trends in retinopathy grading and survival status through severity analysis, and to clarify the potential of retinopathy as an independent indicator of prognostic risk assessment.

## 2. Materials and methods

### 2.1. Sample and study population

Considering the availability of retinal images, we used data sets from two NHANES cycles (2005–2006 and 2007–2008) for this study, led by the National Center for Health Statistics of the US Centers for Disease Control and Prevention (CDC) program. NHANES used a stratified multistage sampling design with a nationally representative survey of the civilian non-institutionalized US population to obtain a representative sample of US residents, the details of the sampling and testing methods have been described extensively elsewhere. The publicly available data used in this project were derived from the NHANES program, which was approved by the Ethics Review Board of the National Center for Health Statistics (NCHS), and in which all participants provided written informed consent to participate in the survey and agreed to the use of their data for health-related statistical research with links to vital statistics (e.g., the National Death Index), adhering to the principles of the Declaration of Helsinki (25). Relevant ethical certifications can be found in Supplement B.

### 2.2. Retinal examination and retinopathy grading

Retinal imaging examinations were performed using an ophthalmic digital imaging system (retinography) (CR6-45NM; Canon United States) and a digital camera (EOS 10D; Canon United States). Two forty-five-degree non-mydriatic digital retinal images of each eye were obtained for all subjects aged 40 years and older who did not meet any of the exclusion criteria (blindness, ocular infection or having eye shields in both eyes) and were photographed in an almost completely darkened room. All fundus digital images were scored by scorers at the University of Wisconsin Ocular Epidemiology Reading Center in Madison, with at least 2 experienced scorers scoring and any discrepancy was adjudicated by a third adjudicator. The severity of retinopathy was assessed according to the Early Treatment Diabetic Retinopathy Study (ETDRS) grading scale. (26) Specifically, we adopted the standard grading guidelines recommended by NHANES, whereby all participants were categorized into 10–80 levels of retinopathy characteristics based on the worse eye. Retinopathy severity was graded into 4 grades (no retinopathy, mild non-proliferative retinopathy (NPR), moderate to severe NPR, and proliferative retinopathy (PR)). The grading of retinopathy is independent of diabetic status and retinopathy may be found in both diabetic and non-diabetic patients. Please refer to the NHANES Ophthalmic Procedures Manual Ophthalmology section (Retinal Imaging (OPXRET_E) OPDDRL4) on the NHANES website for specific grading criteria.

### 2.3. Mortality data

The mortality data in this study were determined using a probabilistic matching algorithm with the National Death Index (NDI) file based on the 2019 Public Access Link mortality file. (27) Specifically, personally identifiable information (e.g., name, sex, date of birth, etc.) was matched between the NHANES and NDI datasets for all NHANES participants aged 18 years or older, and participants who did not match the death certificate or were not identified as dead were considered alive. Under the International Statistical Classification of Diseases and Related Health Problems, Tenth Revision (ICD-10), codes C00-C97 were identified as cancer-specific causes of death. Codes I00-I09, I11, I13, I20-I51 (heart disease), and I60-I69 (cerebrovascular disease) were identified as CVD-specific causes of death, and codes E10-E14 were identified as DM-specific causes of death. The remaining deaths that were not categorized were considered as other causes of death. Follow-up time was calculated based on the time between the date of interview at baseline and the date of death or the end date of the study review (31 December 2019), whichever came first.

### 2.4. Assessment of participant characteristics and covariates

Information on demographic characteristics, health-related behaviors, and comorbidity characteristics was obtained through interviews or physical examinations (physiological measurements, laboratory tests, etc.). Specifically, age was divided into five groups: 40–49, 50–59, 60–69, 70–79, and 80 years or older. Sex was classified as male and female. Ethnicity is divided into four groups: Non-Hispanic White, Non-Hispanic Black, Mexican American, and other. Educational attainment was divided into two groups: below and above, based on whether they had obtained a high school diploma. Marital status is classified as married or living with a partner, and unmarried or other. Economic status was classified as below the poverty line (< 1.00) or at or above the poverty line (≥ 1.00) based on the poverty income ratio (PIR). Smoking status was categorized as never, former, or current. Drinking status was categorized as never, former, current heavy use (≥ 3 drinks per day for females, or ≥ 4 drinks per day for males, or binge drinking on 5 or more days per month), current moderate use (≥ 2 drinks per day for females, or ≥ 3 drinks per day for males, or binge drinking ≥2 days per month) and current mild use (not meet the above criteria). Body mass index (BMI) is defined as weight in kilograms divided by height in meters and is divided into three groups: normal to overweight (18.5–30.0), underweight (< 18.5), and obese (≥ 30.0). DM is defined as doctor told you have DM, or glycohemoglobin HbA1c (%) > 6.5, fasting glucose (mmol/L) ≥ 7.0, random blood glucose (mmol/L) ≥ 11.1, two-hour OGTT blood glucose (mmol/L) ≥ 11.1, or use of DM medication or insulin. Hypertension was defined as a self-reported history of hypertension, use of blood pressure lowering medication, or a systolic blood pressure of 140 mm Hg or higher and/or a diastolic blood pressure of 90 mm Hg or higher, based on the lowest of three measurements. Dyslipidemia or hyperlipidemia is defined as including high triglycerides (TG) (TG ≥ 150 mg/dL), and/or high total cholesterol (TC) (TC ≥ 200 mg/dL), and/or low-density lipoprotein cholesterol (LDL-C) ≥ 130 mg/dL, and/or high total cholesterol (TC) (TC ≥ 200 mg/dL), and/or low-density lipoprotein cholesterol (LDL-C) ≥ 130 mg/dL, and/or high-density lipoprotein cholesterol (HDL) < 40 mg/dL (male) or 50 mg/dL (female) (converted to mmol/L, multiplied by 0.0259), or use of lipid-lowering drugs. High C-reactive protein level (CRP) was defined as a CRP level of at least 1 mg/dL. Depressive symptoms were assessed based on a score of 9-item Patient Health Questionnaire (PHQ-9), with a score greater than or equal to 10 being considered as having depressive symptoms. (28) Difficulty walking was defined as self-reported questionnaire responses or the need for special equipment to assist with walking. Self-rated health status was categorized as poor to fair and good to excellent. A history of systemic comorbidities included physician-diagnosed congestive heart failure, coronary heart disease, angina, heart attack, stroke, and cancer. Age-related ocular comorbidities included cataracts, AMD, and glaucoma, and the associated diagnoses were based on questionnaires and/or retinal image assessment, the specific diagnostic criteria were consistent with previous studies. (29–31)

### 2.5. Statistical analysis

We combined data from two NHANES cycles (2005–2006 and 2007–2008) and conducted all analyses according to the complex stratification design provided by the NHANES Analytics and Reporting Guide. Means and SEs were used for continuous variables, and numerical and weighted percentages were used for categorical variables, to describe the baseline characteristics of all participants (including matched baseline characteristics after propensity score matching (PSM)). These included: age and sex, race, education, marital status, PIR, smoking and drinking status, DM, hypertension and hyperlipidemia, BMI, high CRP levels, depressive symptoms, difficulty walking, self-rated health status, ocular comorbidities (cataracts, AMD, glaucoma) and systemic disease comorbidities (cataracts, AMD, glaucoma), and systemic disease comorbidities (congestive heart failure, coronary heart disease, angina, heart attack, stroke). Unpaired t-tests after design-adjusted and Rao-Scott Pearson χ^2^ tests were used to compare the distribution of continuous or categorical variable data and mortality characteristics, respectively.

Firstly, we used the Kaplan–Meier estimation method to generate survival profiles for participants with retinopathy (dichotomous) as well as severity (quadratus). To identify baseline characteristics associated with survival endpoints, we used age– and sex-adjusted Cox proportional risk regression models to estimate hazard ratios (HRs) and 95% confidence intervals (CIs) for survival. The final Cox proportional risk regression model incorporated covariates significantly associated with both mortality and retinopathy, and HRs were calculated for retinopathy (dichotomous versus quadratic) and mortality. The results of the interaction test suggested no statistically significant interactions between the covariates (*p* > 0.05). The proportional risk hypothesis (PH) was confirmed for each covariate by testing the interaction of each covariate with follow-up time and by graphical representation (*p* > 0.05). To address this competing risk bias, we estimated the risk of cause-specific mortality using a multiple-adjusted Fine and Gray competing risk regression model, given that deaths from other causes can be considered the competing risk event for one specific cause of death. (30, 32)

Sensitivity analyses were performed by adjusting for age and age squared in the final model (Cox proportional risk regression model or Fine and Gray competing risk regression model) to assess the non-linear relationship between age and mortality. (30) The variance inflation factor (VIF) was used to test for covariate effects between all covariates, all of which in this study had VIFs less than 2 [mean (SE), 1.24 (0.07)].

To adequately address the effects of confounding, we further used the PSM approach to control the imbalance of covariates between groups in observational studies. The PSM procedure is effective in reducing confounding bias and provides a similar effect of randomized controlled studies throughout the study design phase, allowing for more reasonable comparisons between different observation groups. (33–35) To control the non-random allocation of included participants, we used the ‘MatchIt’ package in R software to match participants in the retinopathy and non-retinopathy groups on a 1:1 propensity score. Baseline characteristics of all participants were used as matching variables. Comparing baseline information between the two groups of participants before and after PSM, the differences in covariates between the two groups after PSM were found non-statistically significant, indicating that the two groups have achieved relative equilibrium and comparability (eTable 1 in the Supplement A), to compare all-cause and cause-specific mortality between the two groups.

All data analyses were performed using the R version 4.2.1 (2022-06-23) packages “nhanesR,” “survey,” “reshape2 “, “do,” “dplyr” and other packages for statistical analysis. *p*-values less than 0.05 were considered statistically significant differences.

## 3. Results

In the 2005–2008 NHANES, a total of 6,797 participants aged 40 years or older were analyzed. Of these, 1093 participants (16.08%) were excluded due to the absence of graded retinal images, and an additional 896 participants (13.17%) were excluded due to a lack of baseline characteristic information. Ultimately, 4,808 NHANES participants (70.74%) were included in the analysis (eFigure in the Supplement A), representing 18, 282, 772 US non-institutionalized residents. Characteristics of excluded and included participants were compared at baseline, suggesting that included participants were younger [≥80 years, 356 (4.58) vs. 391 (15.02); *p* < 0.0001] and more likely to be non-hispanic white (2,660 (78.54) vs. 886 (66.26); *p* < 0.0001). For other baseline characteristics see eTable 2 in the Supplement A. Table 1 presents the demographic characteristics, health-related behavioral, comorbidities and other general health characteristics of the participants, based on the presence and severity of retinopathy. The results show that participants with retinopathy had a higher proportion of males [341 (58.03%) vs. 249 (41.97%)], tended to be older [≥80 years, 56 (7.79) vs. 300 (4.25)], non-hispanic black [171 (14.85) vs. 785 (8.40)], below high school education [224 (24.49) vs. 1116 (16.31)], smoker [304 (42.99) vs. 773 (13.77)] and former alcohol user [200 (29.94) vs. 1027 (19.96)]; to be obese [257 (44.34) vs. 1581 (36.42)] to have DM [304 (42.99) vs. 773 (13.77)] and hypertension [411 (59.72) vs. 2205 (47.44)], to have difficulty walking (80 (12.77) vs. 378 (6.83)), poor to fair health status (225 (27.48) vs. 992 (16.69)), to have comorbid CVD [e.g., coronary heart disease, 60 (9.36) vs. 212 (4.27)] and comorbid ocular diseases [188 (27.49) vs. 968 (18.16)]. When considering the severity of retinopathy, the above-mentioned differences in baseline characteristic trends (*p* for trend) are still present. Notably, to have high CRP levels and depressive symptoms did not differ significantly between participants with or without retinopathy, but there was a statistically significant difference in trend change between participants with different severities of retinopathy. Other baseline characteristics were not different between groups with and without retinopathy, neither between different severity of retinopathy.

**Table 1:** Demographic, health behavior, and general health characteristics of participants by retinopathy status and grading ^a^.

Characteristic	Study participants							
	All	No retinopathy	Retinopathy	*p* Value	Mild NPR	Moderate to severe NPR	PR	*p* for trend
	*n* ^b^= 4,808 *N* ^c^= 18,282,772	*n* = 4218 *N* = 16,543,908	*n* = 590 *N* = 1,738,864		*n* = 491 *N* = 1,519,059	*n* = 78 *N* = 183,191	*n* = 21 *N* = 36,613	
Age, No. (%), y				< 0.0001				< 0.0001
40–49	1303 (27.1)	1201 (36.34)	102 (22.94)		88 (23.77)	13 (19.42)	1 (5.99)	
50–59	1150 (23.92)	1021 (30.29)	129 (30.18)		109 (30.02)	17 (35.58)	3 (9.79)	
60–69	1210 (25.17)	1018 (18.21)	192 (23.90)		148 (22.97)	28 (20.18)	16 (81.06)	
70–79	789 (16.41)	678 (10.92)	111 (15.20)		95 (15.25)	15 (17.21)	1 (3.17)	
≥80	356 (7.4)	300 (4.25)	56 (7.79)		51 (8.00)	5 (7.61)	0 (0.00)	
Sex, No. (%)				< 0.0001				< 0.0001
Male	2,417 (50.27)	2076 (46.70)	341 (58.03)		286 (58.12)	45 (55.14)	10 (68.75)	
Female	2391 (49.73)	2142 (53.30)	249 (41.97)		205 (41.88)	33 (44.86)	11 (31.25)	
Race/Ethnicity, No. (%)				< 0.0001				< 0.0001
Non-Hispanic White	2,660 (55.32)	2399 (79.34)	261 (70.93)		233 (72.35)	25 (63.07)	3 (51.05)	
Non-Hispanic Black	956 (19.88)	785 (8.40)	171 (14.85)		131 (13.49)	31 (22.68)	9 (31.80)	
Mexican American	731 (15.2)	626 (5.06)	105 (6.96)		83 (6.36)	18 (11.83)	4 (7.34)	
Other	461 (9.59)	408 (7.21)	53 (7.27)		44 (7.79)	4 (2.42)	5 (9.81)	
Marital status, No. (%)				0.56				0.43
Unmarried or other	1712 (35.61)	1493 (30.27)	219 (31.53)		176 (30.89)	31 (33.60)	12 (48.15)	
Married or living with a partner	3096 (64.39)	2725 (69.73)	371 (68.47)		315 (69.11)	47 (66.40)	9 (51.85)	
Educational attainment, No. (%)				< 0.0001				< 0.0001
< High school	1340 (27.87)	1116 (16.31)	224 (24.49)		175 (22.85)	36 (31.29)	13 (58.23)	
≥High school	3468 (72.13)	3102 (83.69)	366 (75.51)		316 (77.15)	42 (68.71)	8 (41.77)	
Poverty income ratio, No. (%)				0.45				0.12
Below poverty line (<1.00)	724 (15.06)	627 (8.52)	97 (9.37)		74 (8.45)	17 (15.94)	6 (14.87)	
At or above poverty line (≥1.00)	4084 (84.94)	3591 (91.48)	493 (90.63)		417 (91.55)	61 (84.06)	15 (85.13)	
Smoking status, No. (%)				< 0.0001				< 0.0001
No	3731 (77.6)	3445 (86.23)	286 (57.01)		277 (63.93)	9 (11.02)	0 (0.00)	
Yes	1077 (22.4)	773 (13.77)	304 (42.99)		214 (36.07)	69 (88.98)	21 (100.00)	
Alcohol consumption, No. (%)				< 0.0001				< 0.001
Never	656 (13.64)	555 (10.34)	101 (16.68)		79 (15.96)	19 (24.07)	3 (9.49)	
Former	1227 (25.52)	1027 (19.96)	200 (29.94)		158 (28.37)	30 (37.13)	12 (58.83)	
Current Mild	1688 (35.11)	1525 (40.96)	163 (32.61)		143 (33.40)	17 (27.76)	3 (24.03)	
Current Moderate	643 (13.37)	586 (15.75)	57 (9.80)		51 (10.71)	4 (3.27)	2 (4.64)	
Current Heavy	594 (12.35)	525 (12.98)	69 (10.98)		60 (11.56)	8 (7.77)	1 (3.01)	
BMI, No. (%)				0.01				0.01
18.5–30.0	2907 (60.46)	2578 (62.36)	329 (55.21)		283 (57.55)	19 (24.07)	7 (27.41)	
<18.5	63 (1.31)	59 (1.22)	4 (0.44)		4 (0.51)	30 (37.13)	0 (0.00)	
≥30.0	1838 (38.23)	1581 (36.42)	257 (44.34)		204 (41.94)	17 (27.76)	14 (72.59)	
High C-reactive protein level, No. (%)				0.67				< 0.0001
No	4237 (88.12)	3719 (89.11)	518 (89.84)		440 (91.66)	65 (83.37)	13 (46.64)	
Yes	571 (11.88)	499 (10.89)	72 (10.16)		51 (8.34)	13 (16.63)	8 (53.36)	
Diabetes mellitus, No. (%)				< 0.0001				< 0.0001
No	3731 (77.6)	3445 (86.23)	286 (57.01)		277 (63.93)	9 (11.02)	0 (0.00)	
Yes	1077 (22.4)	773 (13.77)	304 (42.99)		214 (36.07)	69 (88.98)	21 (100.00)	
Hypertension, No. (%)				< 0.0001				< 0.0001
No	2192 (45.59)	2013 (52.56)	179 (40.28)		161 (42.45)	16 (29.85)	2 (2.43)	
Yes	2616 (54.41)	2205 (47.44)	411 (59.72)		330 (57.55)	62 (70.15)	19 (97.57)	
Hyperlipidemia, No. (%)				0.49				0.53
No	936 (19.47)	836 (19.63)	100 (17.91)		89 (18.40)	9 (16.39)	2 (4.91)	
Yes	3872 (80.53)	3382 (80.37)	490 (82.09)		402 (81.60)	69 (83.61)	19 (95.09)	
Depressive symptoms, No. (%)				0.55				0.04
No	4424 (92.01)	3875 (93.03)	549 (93.95)		459 (95.09)	70 (83.82)	20 (97.17)	
Yes	384 (7.99)	343 (6.97)	41 (6.05)		32 (4.91)	8 (16.18)	1 (2.83)	
Difficulty walking, No. (%)				< 0.001				< 0.0001
No	4350 (90.47)	3840 (93.17)	510 (87.23)		435 (88.87)	62 (78.59)	13 (62.32)	
Yes	458 (9.53)	378 (6.83)	80 (12.77)		56 (11.13)	16 (21.41)	8 (37.68)	
Health status, No. (%)				< 0.0001				< 0.0001
Poor to fair	1217 (25.31)	992 (16.69)	225 (27.48)		168 (24.93)	44 (44.88)	13 (46.30)	
Good to excellent	3591 (74.69)	3226 (83.31)	365 (72.52)		323 (75.07)	34 (55.12)	8 (53.70)	
History of congestive heart failure, No. (%)				< 0.0001				< 0.0001
No	4605 (95.78)	4071 (97.47)	534 (92.25)		453 (93.45)	68 (88.05)	13 (63.06)	
Yes	203 (4.22)	147 (2.53)	56 (7.75)		38 (6.55)	10 (11.95)	8 (36.94)	
History of coronary heart disease, No. (%)				< 0.0001				< 0.0001
No	4536 (94.34)	4006 (95.73)	530 (90.64)		443 (90.73)	72 (91.50)	15 (82.60)	
Yes	272 (5.66)	212 (4.27)	60 (9.36)		48 (9.27)	6 (8.50)	6 (17.40)	
History of angina, No. (%)				< 0.0001				< 0.0001
No	4622 (96.13)	4071 (97.13)	551 (94.08)		458 (93.78)	76 (96.97)	17 (91.69)	
Yes	186 (3.87)	147 (2.87)	39 (5.92)		33 (6.22)	2 (3.03)	4 (8.31)	
History of heart attack, No. (%)				0.001				0.003
No	4522 (94.05)	3993 (95.75)	529 (92.37)		442 (92.36)	71 (92.84)	16 (90.53)	
Yes	286 (5.95)	225 (4.25)	61 (7.63)		49 (7.64)	7 (7.16)	5 (9.47)	
History of stroke, No. (%)				< 0.0001				< 0.0001
No	4564 (94.93)	4036 (96.66)	528 (90.75)		445 (91.35)	67 (88.58)	16 (76.59)	
Yes	244 (5.07)	182 (3.34)	62 (9.25)		46 (8.65)	11 (11.42)	5 (23.41)	
History of cancer, No. (%)				0.57				0.67
No	4209 (87.54)	3690 (88.00)	519 (88.99)		429 (88.53)	72 (92.35)	18 (91.34)	
Yes	599 (12.46)	528 (12.00)	71 (11.01)		62 (11.47)	6 (7.65)	3 (8.66)	
History of comorbid ocular diseases, No. (%)				< 0.001				< 0.0001
No	3652 (75.96)	3250 (81.84)	402 (72.51)		350 (75.14)	46 (61.04)	6 (20.87)	
Yes	1156 (24.04)	968 (18.16)	188 (27.49)		141 (24.86)	32 (38.96)	15 (79.13)	

NPR, Non-proliferative retinopathy; PR, proliferative retinopathy; BMI, body mass index (calculated as weight in kilograms divided by height in meters squared).

a All proportions, means, and SEs are weighted estimates of the US population characteristics, taking into account the complex sampling design of the National Health and Nutrition Examination Survey (NHANES).

b *n* represents the unweighted participant sample size.

c *N* represents the representative population of non-institutionalized residents in the United States weighted according to the complex sampling design of NHANES.

### 3.1. All-cause mortality

Of the 4,808 participants in the current study, 590 (12.27%) had retinopathy at baseline examination, with 491 (10.21%) having mild NPR, 78 (1.62%) moderate to severe NPR, and 21 (0.44%) PR. After a median follow-up of 12.24 years (interquartile range, 11.16–13.49 years), 1,164 participants had died of all-cause mortality, with 941 (80.84%) of these deaths occurring among those without retinopathy and 223 (19.16%) among those with retinopathy at baseline, further classified by the severity of which 172 (14.78%) were mild NPR, 35 (3.01%) were moderate to severe NPR and 16 (1.37%) were PR. The mean (SE) age among non-retinopathy participants [55.73 (0.39) years] at death was significantly younger than that of participants with any retinopathy [59.61 (0.84) years], mild NPR [59.43 (0.90) years], moderate to severe NPR [60.40 (1.51) years], or PR [63.04 (1.41) years] (Table 2). Mortality was significantly higher in participants with mild NPR [172 (31.06%)], moderate to severe NPR [35 (44.63%)], PR [16 (67.21%)] and arbitrary retinopathy [223 (33.25%)] compared to participants without retinopathy [941 (15.72)] (Table 2). In addition, the mean time to death was significantly longer among non-retinopathy participants [145.28 (0.93) months] compared to participant with any retinopathy [131.12 (2.64) months], mild NPR [132.91 (2.43) months], moderate to severe NPR [123.69 (5.85) months], and PR [93.97 (27.59) months] (Table 2).

**Table 2:** Mortality characteristics overall and by different retinopathy status and grading ^a^.

Characteristics	Retinopathy status and grading^b^						
	All	No retinopathy	Any retinopathy	Mild NPR	Moderate to severe NPR	PR	*p* for trend
	*n*^c^= 4,808 N^d^= 18,282,772	*n* = 4218 *N* = 16,543,908	*n* = 590 *N* = 1,738,864	*n* = 491 *N* = 1,519,059	*n* = 78 *N* = 183,191	*n* = 21 *N* = 36,613	
Age at death, mean (SE), y							
Due to all causes	56.10 (0.40)	55.73 (0.39)	59.61 (0.84)^****^	59.43 (0.90)^****^	60.40 (1.51)^**^	63.04 (1.41)^****^	< 0.0001
Due to cancer	65.44 (0.85)	65.06 (0.89)	68.37 (1.95)	69.91 (2.00)^*^	62.16 (2.62)	58.83 (1.72)^**^	0.002
Due to CVD	70.38 (0.60)	70.57 (0.68)	69.68 (1.20)	70.07 (1.13)	69.48 (3.42)	58.01 (4.72)^*^	0.09
Due to DM	63.85 (2.35)	60.60 (3.06)	68.11 (2.55)	71.77 (3.27)^*^	64.25 (2.65)	60.27 (0.00)	0.07
Due to other causes	69.23 (0.83)	69.57 (0.94)	67.52 (1.58)	68.03 (1.77)	64.51 (4.86)	66.22 (0.78)^*^	0.1
Mortality rate, No. (%)							
Due to all causes	1164 (24.21)	941 (15.72)	223 (33.25)^****^	172 (31.06)^****^	35 (44.63)^****^	16 (67.21)^**^	< 0.0001
Due to cancer	279 (5.8)	240 (4.04)	39 (5.04)	31 (4.70)	5 (7.29)	3 (7.81)^*^	0.07
Due to CVD	433 (9.01)	338 (5.62)	95 (14.72)^****^	75 (14.26)^****^	14 (17.79)^****^	6 (18.28)^**^	< 0.0001
Due to DM	40 (0.83)	21 (0.38)	19 (2.76)^****^	13 (1.86)^****^	4 (7.11)^****^	2 (18.44)^****^	< 0.0001
Due to other causes	412 (8.57)	342 (5.69)	70 (10.73)^***^	53 (10.24)^***^	12 (12.44)^**^	5 (22.68)^*^	< 0.0001
Time to death from baseline examination, mean (SE), mo							
Due to all causes	143.93 (0.95)	145.28 (0.93)	131.12 (2.64)^****^	132.91 (2.43)^****^	123.69 (5.85)^***^	93.97 (27.59)	< 0.0001
Due to cancer	87.19 (3.74)	89.45 (4.08)	69.97 (5.80)^*^	70.85 (6.64)^*^	63.64 (14.87)	77.58 (18.36)	0.04
Due to CVD	88.04 (2.34)	89.56 (2.88)	82.49 (3.82)	83.78 (4.35)	77.38 (10.46)	65.59 (16.68)	0.29
Due to DM	97.26 (10.00)	97.05 (10.67)	97.55 (16.69)	89.39 (21.53)	113.66 (17.53)	100.60 (0.00)	0.56
Due to other causes	92.67 (3.20)	93.17 (3.04)	90.14 (6.23)	95.60 (6.91)	74.51 (7.99)^*^	30.75 (21.37)^*^	0.05

NPR, Non-proliferative retinopathy; PR, proliferative retinopathy; CVD, cardiovascular disease; DM, Diabetes mellitus.

a Mortality was assessed through December 31, 2020. All proportions, means, and SEs are weighted estimates of the US population characteristics, taking into account the complex sampling design of the National Health and Nutrition Examination Survey (NHANES).

b All *p* values were calculated using the unpaired t test for continuous variables and the design-adjusted Rao-Scott Pearson χ^2^ test for categorical variables. Comparisons were between each group with retinopathy and the group with no retinopathy and were unadjusted.

c *n* represents the unweighted participant sample size.

d *N* represents the representative population of non-institutionalized residents in the United States weighted according to the complex sampling design of NHANES.

*p < 0.05; **p < 0.01; ***p < 0.001; ****p < 0.0001.

Table 3 presents the correlation between baseline covariates and all-cause mortality as determined by age- and sex-adjusted Cox proportional hazard regression models. It is worth noting that the HRs increase exponentially with each decade of age. Women have a lower risk of all-cause mortality (HR = 0.68; 95% CI: 0.61–0.76), and non-hispanic black have a higher risk of all-cause mortality (HR = 1.45; 95% CI:1.25–1.69), In addition, the covariates including education (HR = 0.60; 95% CI:0.51–0.70), marital status (HR = 0.60; 95% CI:0.54–0.67), family income (HR = 0.47; 95% CI:0.39–0.57), smoking status (HR = 1.67; 95% CI:1.45–1.92), alcohol consumption (HR = 0.57; 95% CI:0.45–0.74), DM (HR = 1.67; 95% CI:1.45–1.92) and hypertension (HR = 1.34; 95% CI:1.15–1.56), BMI (HR = 2.79; 95% CI:1.36–5.73), high CRP level (HR = 1.93; 95% CI:1.46–2.56), depressive symptoms (HR = 2.08; 95% CI:1.64–2.64), self-rated health status (HR = 0.46; 95% CI:0.40–0.53), difficulty walking (HR = 2.66; 95% CI:2.25–3.15), and self-reported history of CVD (e.g., for coronary heart disease, HR = 1.47; 95% CI:1.22–1.76) or cancer (HR = 1.22; 95% CI:1.06–1.40) and history of comorbid ocular diseases (HR = 1.44; 95% CI:1.24–1.67) were significantly associated with an increased risk of all-cause mortality.

**Table 3:** Due to all causes mortality by demographic, health-related behaviors and general health characteristics b^a^.

Characteristics	Participants		
	Survived (*n* ^b^=3644) *N* ^c^= 15,103,477	Died (*n* = 1164) *N* = 3,179,295	HR (95% CI)^d^
Age, No. (%), y			
40–49	1233 (40.68)	70(8.38)	1 [Reference]
50–59	1026 (33.70)	124(14.01)	1.99 (1.36 to 2.92)^***^
60–69	920 (17.60)	290(24.19)	6.07 (4.72 to 7.82)^****^
70–79	399 (6.94)	390(32.17)	16.21 (11.89 to 22.09)^****^
≥80	66 (1.08)	290(21.25)	42.51 (32.00 to 56.48)^****^
Sex, No. (%)			
Male	1739 (46.86)	678 (52.14)	1 [Reference]
Female	1905 (53.14)	486 (47.86)	0.68 (0.61 to 0.76)^****^
Race/Ethnicity, No. (%)			
Non-Hispanic White	1883 (77.53)	777 (83.36)	1 [Reference]
Non-Hispanic Black	721 (8.77)	235 (10.15)	1.45 (1.25 to 1.69)^****^
Mexican American	650 (5.78)	81 (2.64)	0.72 (0.58 to 0.90)^**^
Other	390 (7.92)	71 (3.85)	0.77 (0.49 to 1.21)
Marital status, No. (%)			
Unmarried or other	1180 (27.50)	532 (44.11)	1 [Reference]
Married or living with a partner	2464 (72.50)	632 (55.89)	0.60 (0.54 to 0.67)^****^
Educational attainment, No. (%)			
<High school	915 (14.22)	425 (30.70)	1 [Reference]
≥High school	2729 (85.78)	739 (69.30)	0.60 (0.51 to 0.70)^****^
Poverty income ratio, No. (%)			
Below poverty line (<1.00)	504 (7.55)	220 (13.61)	1 [Reference]
At or above poverty line (≥1.00)	3140 (92.45)	944 (86.39)	0.47 (0.39 to 0.57)^****^
Smoking status, No. (%)			
No	2946 (86.19)	785 (70.44)	1 [Reference]
Yes	698 (13.81)	379 (29.56)	1.67 (1.45 to 1.92)^****^
Alcohol consumption, No. (%)			
Never	457 (9.67)	199 (16.99)	1 [Reference]
Former	808 (18.18)	419 (33.87)	1.20 (0.94 to 1.52)
Mild	1340 (42.16)	348 (30.72)	0.57 (0.45 to 0.74)^****^
Moderate	535 (16.13)	108 (10.68)	0.81 (0.58 to 1.13)
Heavy	504 (13.85)	90 (7.73)	0.89 (0.68 to 1.16)
BMI, No. (%)			
18.5–30.0	2147 (61.10)	760 (64.44)	1 [Reference]
<18.5	36 (0.94)	27 (2.10)	2.79 (1.36 to 5.73)^**^
≥30.0	1461 (37.96)	377 (33.46)	1.03 (0.91 to 1.17)
High C-reactive protein level, No. (%)			
No	3260 (90.20)	977 (84.33)	1 [Reference]
Yes	384 (9.80)	187 (15.67)	1.93 (1.46 to 2.56)^****^
Diabetes mellitus, No. (%)			
No	2946 (86.19)	785 (70.44)	1 [Reference]
Yes	698 (13.81)	379 (29.56)	1.67 (1.45 to 1.92)^****^
Hypertension, No. (%)			
No	1847 (55.73)	345 (30.79)	1 [Reference]
Yes	1797 (44.27)	819 (69.21)	1.34 (1.15 to 1.56)^***^
Hyperlipidemia, No. (%)			
No	713 (19.76)	223 (18.07)	1 [Reference]
Yes	2931 (80.24)	941 (81.93)	0.85 (0.70 to 1.02)
Depressive symptoms, No. (%)			
No	3363 (93.61)	1061 (90.76)	1 [Reference]
Yes	281 (6.39)	103 (9.24)	2.08 (1.64 to 2.64)^****^
Difficulty walking, No. (%)			
No	3432 (95.53)	918 (78.67)	1 [Reference]
Yes	212 (4.47)	246 (21.33)	2.66 (2.25 to 3.15)^****^
Health status, No. (%)			
Poor to fair	798 (14.69)	419 (32.11)	1 [Reference]
Good to excellent	2846 (85.31)	745 (67.89)	0.46 (0.40 to 0.53)^****^
History of congestive heart failure, No. (%)			
No	3573 (98.82)	1032 (88.19)	1 [Reference]
Yes	71 (1.18)	132 (11.81)	3.35 (2.76 to 4.07)^****^
History of coronary heart disease, No. (%)			
No	3519 (96.92)	1017 (87.32)	1 [Reference]
Yes	125 (3.08)	147 (12.68)	1.47 (1.22 to 1.76)^****^
History of angina, No. (%)			
No	3552 (97.90)	1070 (91.80)	1 [Reference]
Yes	92 (2.10)	94 (8.20)	1.45 (1.14 to 1.85)^**^
History of heart attack, No. (%)			
No	3525 (97.27)	997 (86.65)	1 [Reference]
Yes	119 (2.73)	167 (13.35)	1.93 (1.57 to 2.39)^****^
History of stroke, No. (%)			
No	3538 (97.77)	1026 (88.17)	1 [Reference]
Yes	106 (2.23)	138 (11.83)	2.08 (1.74 to 2.49)^****^
History of cancer, No. (%)			
No	3313 (90.39)	896 (77.17)	1 [Reference]
Yes	331 (9.61)	268 (22.83)	1.22 (1.06 to 1.40)^**^
History of comorbid ocular diseases, No. (%)			
No	3051 (86.74)	601 (53.46)	1 [Reference]
Yes	593 (13.26)	563 (46.54)	1.44 (1.24 to 1.67)^****^

Abbreviations: NPR, Non-proliferative retinopathy; PR, proliferative retinopathy; BMI, body mass index (calculated as weight in kilograms divided by height in meters squared); HR, hazard ratio; CI, confidence interval.

a All-cause mortality was assessed through December 31, 2020. All proportions, means, and SEs are weighted estimates of the US population characteristics, taking into account the complex sampling design of the National Health and Nutrition Examination Survey (NHANES).

b n represents the unweighted participant sample size.

c N represents the representative population of non-institutionalized residents in the United States weighted according to the complex sampling design of NHANES.

d Adjusted for age and sex.

^*^p < 0.05; ^**^p < 0.01; ^***^p < 0.001; ^****^p < 0.0001.

**Figure 1:**
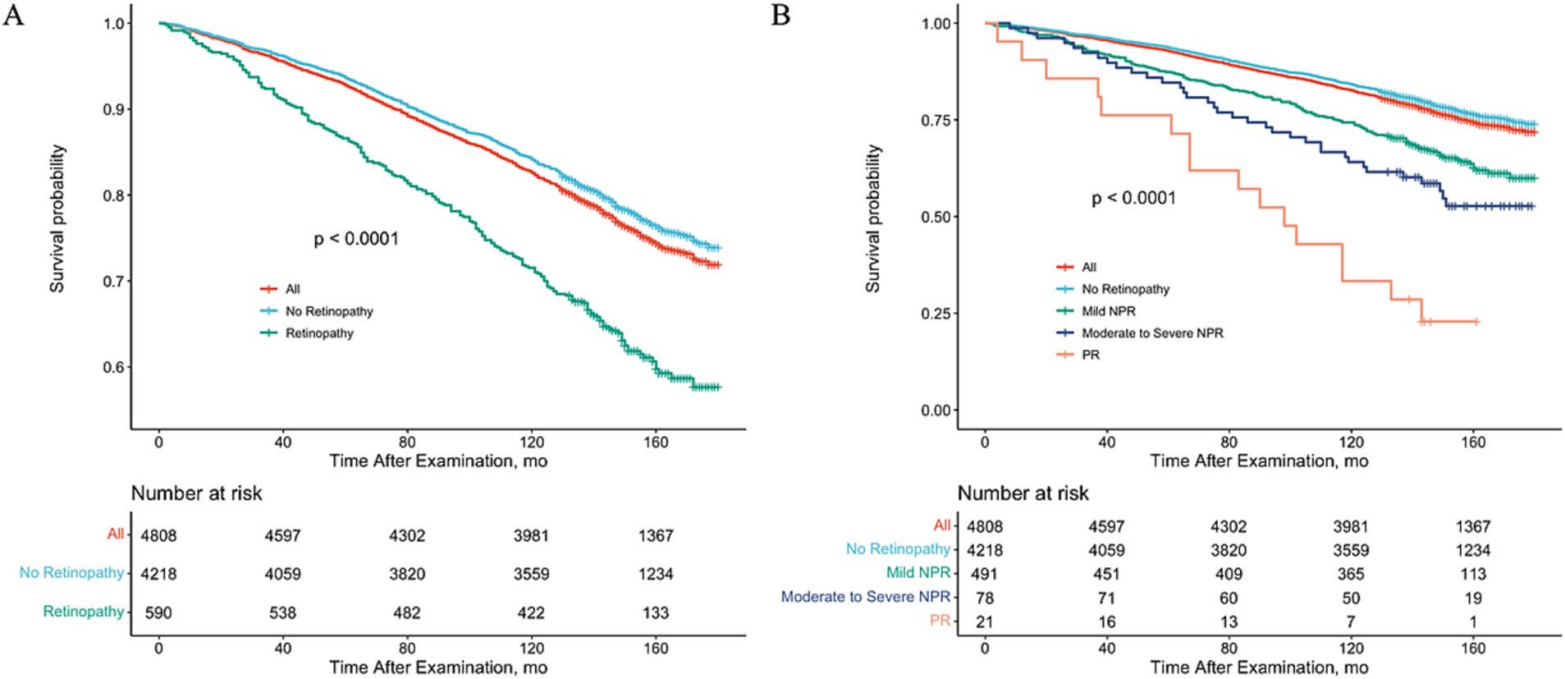
Adjusted Kaplan–Meier Curve for All-Cause Mortality Rate by Retinopathy Status. Study results were stratified according to the presence or absence of retinopathy **(A)** or retinopathy grading **(B)**, using 2005–2008 National Health and Nutrition Examination Survey (NHANES) data. All-cause mortality was assessed through 31 December 2020.

After controlling for variables significantly associated with mortality and retinopathy, multivariate Cox regression models showed that poorer survival was associated with any retinopathy (HR = 2.01; 95% CI, 1.00–4.03) and mild NPR (HR = 2.01; 95% CI, 1.00–4.03) compared to participants without retinopathy at baseline (Table 4). However, participants with moderate to severe NPR and PR did not present a higher mortality risk, compared with those without retinopathy at baseline, but there was a significant difference in the trend of greater risk of mortality as the severity of retinopathy increased (*p* for trend = 0.01) (Table 4). To visually depict these findings, we plotted multiple-adjusted Kaplan–Meier curves for all-cause mortality based on retinopathy status/severity (Figure 1).

**Table 4:** Cox proportional hazards models for all-cause mortality and fine and gray competing risks regression models for specific-cause mortality by retinopathy status.

Retinopathy Status	Mortality									
	Due to all causes *n* ^a^= 1164 *N* ^b^= 3,179,295		Due to cancer *n* = 279 *N* = 755,272		Due to CVD *n* = 433 *N* = 1,185,524		Due to DM *n* = 40 *N* = 111,082		Due to other causes *n* = 412 *N* = 1,127,417	
	HR ^c^ (95% CI)	Squared age adjusted HR ^d^ (95% CI)	HR (95% CI)	Squared age adjusted HR (95% CI)	HR (95% CI)	Squared age adjusted HR (95% CI)	HR (95% CI)	Squared age adjusted HR (95% CI)	HR (95% CI)	Squared age adjusted HR (95% CI)
None	1 [Reference]	1 [Reference]	1 [Reference]	1 [Reference]	1 [Reference]	1 [Reference]	1 [Reference]	1 [Reference]	1 [Reference]	1 [Reference]
Any	1.35 (1.08 to 1.67)^**^	1.34 (1.08 to 1.67)^**^	0.97 (0.69 to 1.37)	0.95 (0.68 to 1.35)	1.64 (1.30 to 2.07)^****^	1.65 (1.30 to 2.08)^****^	5.21 (2.79 to 9.70)^****^	5.19 (2.79 to 9.68)^****^	1.12 (0.86 to 1.47)	1.12 (0.85 to 1.48)
Mild NPR	1.32 (1.05 to 1.66)^*^	1.32 (1.05 to 1.65)^*^	0.85 (0.58 to 1.26)	0.85 (0.58 to 1.24)	1.48 (1.14 to 1.91)^**^	1.48 (1.14 to 1.91)^**^	4.13 (2.00 to 8.54)^***^	4.14 (2.00 to 8.53)^***^	0.97 (0.71 to 1.31)	0.96 (0.71 to 1.30)
Moderate to Severe NPR	1.44 (0.89 to 2.32)	1.43 (0.89 to 2.31)	0.90 (0.37 to 2.19)	0.88 (0.36 to 2.15)	2.02 (1.17 to 3.49)^*^	2.04 (1.18 to 3.51)^*^	6.79 (2.11 to 21.87)^**^	6.77 (2.13 to 21.51)^**^	1.39 (0.73 to 2.63)	1.39 (0.73 to 2.64)
PR	1.83 (0.59 to 5.72)	1.86 (0.58 to 5.92)	1.95 (0.59 to 6.48)	1.72 (0.52 to 5.76)	3.97 (1.71 to 9.19)^**^	4.10 (1.76 to 9.52)^**^	13.62 (2.52 to 73.75)^**^	13.46 (2.50 to 72.38)^**^	3.06 (1.11 to 8.42)^*^	3.23 (1.18 to 8.88)^*^
*P* for trend	0.01	0.01	0.67	0.47	0.004	0.01	0.04	< 0.0001	0.08	0.04

NPR, Non-proliferative retinopathy; PR, proliferative retinopathy; CVD, cardiovascular disease; DM, Diabetes mellitus; HR, hazard ratio; CI, confidence interval.

a n represents the unweighted participant sample size.

b N represents the representative population of non-institutionalized residents in the United States weighted according to the complex sampling design of the National Health and Nutrition Examination Survey (NHANES).

c Adjusted for age, sex, race/ethnicity, educational attainment, marital status, body mass index, family income, smoking status, alcohol consumption, diabetes mellitus, hypertension, high C-reactive protein level, depressive symptoms, walking disability, self-rated health, history of coronary heart disease, congestive heart failure, heart attack, stroke and comorbid ocular diseases.

d Adjusted for age, squared age, sex, race/ethnicity, educational attainment, marital status, body mass index, family income, smoking status, alcohol consumption, diabetes mellitus, hypertension, high C-reactive protein level, depressive symptoms, walking disability, self-rated health, history of coronary heart disease, congestive heart failure, heart attack, stroke and comorbid ocular diseases.

*p < 0.05; **p < 0.01; ***p < 0.001; ****p < 0.0001.

In addition, the cox proportional hazards models were recreated after the PSM with balanced covariates for the two groups of participants matched 1:1. The model included 590 participants with retinopathy, representing the 4,572,861 US non-institutionalized resident population, matched to 590 non-retinopathy patients representing the 4,697,061 US non-institutionalized resident population. Results demonstrate that participants with any retinopathy have a higher risk of all-cause mortality (HR = 1.40; 95% CI, 1.01–1.93) compared to participants without retinopathy (Table 4).

### 3.2. Cause-specific mortality

Among the 1164 participants in the study, 279 (23.97%) died of cancer, 433 (38.06%) died of CVD, 40 (3.44%) died of DM and 412 (35.40%) died of other causes not mentioned above. The results in Table 2 suggest that the presence of any retinopathy and all levels of retinopathy were associated with increased risks of specific-causes of death except cancer, while the increased risk of cancer-caused death was only significantly associated with PR. Results from competing risk regression models for cause-specific mortality suggested that, after multiple adjustment for covariates, participants with any retinopathy and any severity (mild, moderate to severe NPR and PR) of retinopathy were significantly associated with an increased CVD-caused and DM-caused mortality risk compared with participants without retinopathy. Participants with any retinopathy were associated with a nearly 2-fold (HR = 1.64; 95% CI, 1.30–2.07) and more than 5-fold (HR = 5.21; 95% CI, 2.79–9.70) increased CVD-caused and DM-caused mortality risk, respectively. The CVD and DM-specific HRs increased exponentially with increasing severity of retinopathy (for CVD, ranging from 1.48 (95% CI, 1.14 to 1.91) for the mild NPR group to 3.97 (1.71 to 9.19) for the PR group; for DM, ranging from 4.13 [95% CI, 2.00 to 8.54) for the mild NPR group to 13.62 (2.52 to 73.75) for the PR group], with significant trend differences (*p* for trend 0.004 and 0.04, respectively) after multiple adjustments (Table 4).

However, after multivariate adjustment, there was no association between any retinopathy or level of severity of retinopathy and cancer-specific mortality. There was a significant association between PR and other causes specific mortality (HR = 3.06; 95% CI, 1.11–8.42). However, trend tests did not demonstrate their significant differences with retinopathy severity (Table 4).

The results after PSM suggested that participants with any retinopathy had a higher risk of CVD-specific (HR = 1.91; 95% CI, 1.20–3.04) and DM-specific mortality (HR = 2.45; 95% CI, 1.00–6.04) compared to those without retinopathy. However, there was no significant difference with cancer-specific and other causes-specific mortality risks (Table 4).

### 3.3. Sensitivity analyses

We included age squared in the final model (Cox proportional risk regression model or Fine and Gray competing risk regression model) to correct the non-linear relationship between age and mortality for sensitivity analysis, and the results observed were like those presented in the main analysis (Table 4).

## 4. Discussion

In this study, based on the updated NHANES follow-up mortality data, we analyzed a nationally representative sample of 4,808 US adults aged 40 years or older, representing 18, 282, 772 non-institutionalized US residents, and we report that the increased risk of all-cause mortality was only associated with any retinopathy and mild NPR. While the increased risk of CVD-specific and DM-specific mortality was associated with any retinopathy and all levels of retinopathy severity, and conversely, none were associated with cancer-specific mortality. In addition, PR was associated with increased mortality for other (non-cancer, non-CVD, and non-DM) specific causes. Multiple adjustment for the range of traditional risk factors such as CVD comorbidities, hypertension and DM was consistent with the results after PSM. Therefore, retinopathy is a risk factor for all-cause, CVD, and DM-specific mortality, which is independent of CVD comorbidity, DM, hypertension, and other risk factors. Severity analysis shows that, increased severity of retinopathy was significantly associated with increased trends for all-cause, CVD-specific, or DM-specific mortality risk. Specifically, we elucidated that CVD and DM-specific HRs increased exponentially with increasing retinopathy severity, having any retinopathy significantly associated with nearly twice the CVD-specific mortality risk and more than 5-fold the DM-specific mortality risk, respectively.

The results of previous population-based studies on the relationship between retinopathy and mortality are summarized in eTable 3 in the Supplementary A. (17, 21, 23, 24, 36–38) Our findings reconfirm that the presence of any retinopathy is associated with an increased risk of all-cause mortality, which is generally consistent with the findings of previously published population-based studies including the BioBank Japan Cohort, (39) the Age, Gene/Environment Susceptibility-Reykjavik Study (AGES-RS), (21) the Swedish National Cohort Study, (40) one previous NHANES, (24) and the EURODIAB Study. (41) Further, in severity analyses, although we found an increased trend in all-cause mortality significantly associated with increased severity of retinopathy, our analysis showed that only mild NPR was associated with all-cause mortality, and moderate to severe NPR and PR were not associated with all-cause mortality, but were associated with increased CVD and DM-specific mortality. Similarly, after adjusting for all explanatory risk factors, Manon V Van Heck et al. did not find a significant effect of PR on the risk of all-cause mortality in the Hoorn Study (HR = 1.4, 95%CI: 0.9–2.1). (36) However, another population-based cohort study, the Singapore Epidemiology of Eye Diseases study, covering 2964 people aged 40–80 years with DM, reported that moderate NPR (HR = 1.78, 95% CI: 1.26–2.52) and severe NPDR or PDR (HR = 2.75, 95% CI: 1.93–3.92) were significantly associated with a 1.78-fold and 2.75-fold risk of all-cause mortality, respectively. (18) This is supported by the results of a prospective cohort study based on type 2 diabetic patients recruited in 19 Italian centers between 2006 and 2008, which found that patients with advanced retinopathy (including severe NPR and PR) (HR = 1.213) or PR alone (HR = 1.381) both had significantly increased adjusted mortality risks (*p* < 0.0001). (42) However, the results of another Danish cohort study based on type 1 diabetic patients under 30 years of age were different since they reported that, after 25 years of follow-up, compared to patients without retinopathy, patients with NPR (including all degrees of NPR) had a non-significant increased risk of all-cause mortality (HR = 1.01, 95% CI: 0.72–1.42), while patients with PR had a significantly increased risk of death (HR = 2.04, 95% CI: 1.43–2.91) (after adjusting for gender and age). (43)

Although studies of retinopathy and mortality risk have been conducted in other regions, the relationship between retinopathy progression and mortality outcomes in the U.S. population is currently unclear. An analysis by Zhu et al. (24) of the U.S. population over 40 years of age, data from the 2005–2008 National Health and Nutrition Examination Survey, showed that the presence of retinopathy predicted higher all-cause mortality (HR = 1.41, 95% CI: 1.08–1.83) compared to the healthy groups, and another study based on data from the 1988–1994 NHANES database indicated a similar point (HR = 2.39, 95% CI: 1.77–3.22). (17) However, none of them have elucidated the impact of retinopathy severity on mortality, and the relationship between the timing of conducting fundus screenings and patient prognosis remains unclear for U.S. patients with retinopathy, especially diabetic patients with high disease burden, even as a gap remains in the area of early grading and management of the disease.

In conclusion, although a growing number of studies have confirmed serous retinopathy (or PR) in specific disease populations (e.g., diabetics) (44) or retinopathy combined with systemic vascular comorbidities (24, 45, 46) were significantly associated with all-cause mortality, there is still no consensus on the relationship between independent grading of retinopathy and all-cause mortality in the general population under severity analysis. Our study reports the significant association of mild NPR with reduced all-cause survival in the U.S., the exact reason remains unclear, and these inconsistent results may be attributable to differences in the demographic characteristics of the study population, as well as differences in the underlying comorbidities (e.g., DM, chronic kidney disease (CKD), CVD), the criteria for assessment and grading of retinopathy, the number and extent of confounding factors included, and the systematic inaccuracy of the stratified sample.

The results of our cause-specific mortality-based study showed that the presence of retinopathy remained correlated with increased mortality due to CVD after multiple adjustment for confounders or PSM, and the results of the severity analysis confirmed that retinopathy of all grades was associated with CVD-specific causes of death, furthermore, the trend of retinopathy progression was significantly associated with the trend of worsening survival in CVD patients. Our results support several previous studies, with Fisher et al. reporting in the AGES-RS cohort study that multivariate analysis suggested that retinopathy in the older adults was significantly associated with mortality due to CVD (HR = 1.57, 95% CI: 1.20–2.06), and higher when accompanied by a history of stroke (HR = 3.30, 95% CI: 2.05–5.32), etc. (21) In the Ibaraki Prefectural Health Study, Toshimi Sairenchi et al. showed that an increased risk of CVD death in the region over a 14-year period was independently associated with mild hypertensive retinopathy in patients. (38) Another meta-analysis, which included 20 cohort studies, reported that retinopathy was associated with an increased risk of mortality due to CVD in patients with DM. (22) However, our results were contrary to those of the 1988–1994 NHANES (HR = 0.96, 95% CI: 0.50–1.84) (17) and the Hoorn Study (HR = 1.4, 95% CI: 0.7–2.8), (36) which reported an increased risk of CVD in patients with DM, they failed to detect a significant association between retinopathy and CVD-specific mortality after adjusting for multiple confounders. In conclusion, there is inconsistency in the significance and magnitude of the association between retinopathy and CVD-specific mortality, and this inconsistency may be attributable to many factors. First, given that DM is the most important risk factor for CVD patient mortality and that DM-induced retinopathy is the predominant subtype of retinopathy, under-adjustment for DM-related complications, vascular comorbidities and DM duration may overestimate the risk of survival due to CVD in patients with retinal disease. A recent longitudinal study lasting 21 years also showed that individual or combined DM-related microvascular complications (e.g., CKD, DR, etc.) have a significant impact on mortality independent of known confounders. (47) The possible common pathophysiological mechanisms underlying CVD caused by macrovascular adverse events and DR with microangiopathy as the main manifestation may confound the true cause of death in some CVD patients, (36) suggesting further differentiation needs to be explored and confirmed. More importantly, none of the previous studies have considered competing risks of death when estimating specific mortality in the over-40 age group, and the use of Cox proportional risk regression models may lead to an overestimation of the absolute risk of specific mortality We addressed this methodological issue for the first time using a competing risk model adjusted for multiple confounders (including DM), clarifying the significant relationship between retinopathy and CVD-specific mortality risk. Finally, previous studies have not adequately adjusted for important confounders such as underlying medical history (CVD such as coronary artery disease, stroke, myocardial infarction, etc.), ophthalmic co-morbidities, etc., which may have overestimated the significant association between retinopathy and CVD mortality.

Notably, we found that after adjustment for multiple confounders, compared to participants without retinopathy, the presence of any retinopathy was associated with more than 5-fold greater DM-specific mortality, even more than 2-fold after PSM, which had a greater impact on survival attributable to DM than all-causes, CVD, cancer, or other causes. This result is consistent with the findings of the CHAMP1ON study, (47) one previous NHANES study, (24) and the Southern California cohort study. (16) In addition, the results of the severity analysis similarly confirmed that all grades of retinopathy were significant associated with DM-specific causes of death, and the trend test was also significant (*P* for trend <0.05), the DM-specific HR increased exponentially with increasing retinopathy severity, and patients with PR had the highest risk of DM-specific death (HR = 13.62, 95% CI: 2.52–73.75). As for the reasons why grading of retinopathy was associated with the trend of increased DM-specific mortality risk, the results of a multi-ethnic Asian population study showed that survival rates of DM patients without microvascular disease were similar to those of non-DM patients after approximately 7 years, (48) suggesting that the lesion burden of the microvasculature in DM patients predicts an increased mortality risk. As previously described, the retina is the only site where blood vessels are directly visible, and the grading of retinopathy obtained by fundus photography may be an indicator of systemic microvascular disease characteristics, which could explain the fact that increased severity of retinopathy leads to an exponential increase in the DM-specific mortality risk. In addition, given that older people with long-term DM often have other systemic diseases, particularly CKD and CVD, early findings suggest that the joint effect of retinopathy and microproteinuria significantly increases the risk of DM-related mortality. (43) The Susceptibility-Reykjavik Study has also shown that the presence of retinopathy with vascular co-morbidities such as DM, CVD and CKD can further increase the risk of death, (21) since our study found that retinopathy can increase the DM-specific risk of death even after adjusting for CVD-related co-morbidities. Retinopathy, particularly PR, has been shown to be an important indicator of the stage of DM progression, and therefore it can be hypothesized that DM patients with severe retinopathy may also suffer from other serious complications accelerating adverse survival events such as cardio-vascular infarction, cognitive burden, neurological deficits, etc., thus contributing to an increased mortality risk, as supported by Bjerg et al. (40) A meta-analysis of 77 studies from 26 countries including 99,847 patients with newly diagnosed diabetes mellitus type 2 (T2DM) suggested that, a pooled prevalence of 13.1% for retinopathy in newly diagnosed T2DM patients, DR is a prevalent complication in newly diagnosed T2DM patients. (49) In summary, our findings suggest that retinopathy may be one of the strong independent predictors for assessing survival in DM patients, and given the accessibility and accuracy of fundus examination, retinopathy is valuable in regular screening for early diagnosis of DM, and routine ophthalmic assessment after DM diagnosis.

Our results did not suggest a significant association between retinopathy and cancer-specific mortality risk, and the trend test was also not statistically significant, supporting the findings of several published studies. A Japanese cohort study from 2003 to 2007 found that the most common cause of death in the retinopathy group was cancer, but there was no significant difference for cancer mortality between the retinopathy and normal populations. (39) Similarly, Kim et al. did not detect a correlation between vitreoretinal disease and cancer mortality in the older adults. (50)

Given the convenience of obtaining the diagnosis and grading of retinopathy by fundus examination in a non-invasive manner, the findings of our study have several practical implications. Firstly, our current study found that the diagnosis of retinopathy was an independent risk factor for all-cause mortality in people over 40 years, suggesting that the general older adults population may benefit from a routine fundus examination program. Secondly, the grading of retinopathy was significantly associated with the survival risk of patients with CVD or DM, highlighting the value of retinal images as a valid predictor in monitoring the status of systemic macrovascular and microvascular lesions. Finally, we found that increasing severity of retinopathy was significantly associated with an exponential increase in CVD or DM-specific mortality risk; therefore, closely dynamic detection of retinopathy may be beneficial for stratified management and early warning of CVD and DM patients.

Given the ease of obtaining a diagnosis and grading of retinopathy in a noninvasive manner through fundus examination, our findings have several practical implications. First, our current study found that the diagnosis of retinopathy was an independent risk factor for all-cause mortality in people over 40 years of age, suggesting that the general older adult population may benefit from regular fundus exams as well as specialized retinal disease screening programs such as fundus photography, optical coherence tomography (OCT), and slit-lamp exams, with a particular focus on retinopathy-related pathology. Second, retinopathy grading was significantly associated with survival risk in patients with cardiovascular disease or diabetes mellitus, suggesting that patients with cardiovascular disease or diabetes mellitus need to undergo routine fundus examinations and retinal disease screening on a regular basis for easy and rapid monitoring and management of systemic macrovascular and microvascular disease conditions. Finally, we found that an increase in retinopathy severity was significantly associated with an exponential rise in the risk of death specific to cardiovascular disease or diabetes mellitus. Thus, this emphasizes that patients with cardiovascular disease and diabetes should be closely and dynamically monitored for the onset and progression of retinopathy through aggressive and regular fundus examinations to assist physicians in stratified management and early warning.

## 5. Strengths and limitations

The strengths of this study were, firstly, we applied a large, nationally representative sample of older adults with complete updated NHANES follow-up mortality data and a standardized, objective, graded retinopathy assessment protocol. Secondly, none of the previous studies considered competing risks of death in the calculation of specific causes of death, we addressed this methodological issue by using a multiple confounder-adjusted competing risks model to clarify the significant association of retinopathy with CVD and DM-specific mortality risks. In addition, previous studies have insufficiently adjusted for important confounders such as CVD comorbidities and age-related ocular comorbidities, we offered a comprehensive set of demographic characteristics, health-related indicators, comorbidities, and complete mortality records, and provided results for multiple adjusted covariates. The application of the PSM then adequately corrected these confounding characteristics. Finally, we also conducted the trend test for retinopathy severity grading and mortality.

However, several limitations of this study must be considered.

1. Retinopathy status or grading were assessed at the same time as the confounding factors, making it difficult to specifically evaluate changes in retinopathy, health-related behaviors, and comorbid conditions during the follow-up period.
2. Although we have adjusted for a range of traditional and specific confounders, there are still potentially important confounders that might have been missed, such as laser eye surgery, anti-vascular endothelial growth factor (VEGF) treatment.
3. Self-reported interview and clinical examination data were used, which may have led to some mild cases missing, while the NHANES sample design excluded hospitalized patient data, which may have led to some severe cases missing.
4. The participants we excluded were older and worse in comorbidities and health behaviors or characteristics, which may have influenced the analysis of the results. Nevertheless, our after-PSM data adequately balance these differences in baseline characteristics, and the results remain consistent, thus validating the robustness of our conclusions.

## 6. Conclusion

In summary, our findings suggest that the presence of retinopathy is associated with increased all-cause mortality, CVD, and DM-specific mortality in a large nationally representative sample of US older adults non-hospitalized participants with complete updated NHANES follow-up mortality data. Severity analysis showed that only mild NPR was associated with an increased all-cause mortality risk, while the increased CVD and DM-specific mortality was associated with all retinopathy grades, and grew exponentially with increasing retinopathy severity, the trend test also showed significant differences. Our results clarify the relationship between retinopathy and all-cause and specific-cause mortality, highlight the potential of retinopathy as an independent, convenient, continuously measurable, and valid predictor of CVD and DM disease survival status, suggesting its value in monitoring systemic vasculopathy status, as well as its significance in the health management and risk warning of CVD and DM patients.

## Data availability statement

The original contributions presented in the study are included in the article/Supplementary material, further inquiries can be directed to the corresponding authors.

## Ethics statement

The studies involving humans were approved by the Ethics Review Board of the National Center for Health Statistics. The studies were conducted in accordance with the local legislation and institutional requirements. Written informed consent for participation was not required from the participants or the participants’ legal guardians/next of kin in accordance with the national legislation and institutional requirements.

## Author contributions

S-YG conceived and designed the study. J-CQ and X-CW provided methodological support. S-YG, Q-QW, and S-YL were responsible for analyzing the data. D-WL, M-YZ, Z-HH, and Y-YX organized and interpreted the data and drafted the manuscript. L-MT, C-YH, and Z-XJ revised and reviewed the manuscript. All authors have read and agreed to the published version of the manuscript.

## Funding

This article was supported by the National Natural Science Foundation of China (nos. 52202343, 82070986, and 82171043), the Anhui Provincial Natural Science Foundation (2208085QC81), and the Basic and Clinical Cooperative Research and Promotion Program of Anhui Medical University (2021xkjT028).

## Conflict of interest

The authors declare that the research was conducted in the absence of any commercial or financial relationships that could be construed as a potential conflict of interest.

## Publisher’s note

All claims expressed in this article are solely those of the authors and do not necessarily represent those of their affiliated organizations, or those of the publisher, the editors and the reviewers. Any product that may be evaluated in this article, or claim that may be made by its manufacturer, is not guaranteed or endorsed by the publisher.
