# Supplementary material for: Association of retinopathy with risk of all-cause and specific-cause mortality in the National Health and Nutrition Examination Survey, 2005 to 2008
Source: Front Public Health. 2023 Aug 23;11:1200925. doi: 10.3389/fpubh.2023.1200925 (PMC10482412; doi:10.3389/fpubh.2023.1200925)
Supplement: Supplementary file 1 [file Table_1.DOCX]

Supplementary Material A

Association of Retinopathy with Risk of All-Cause and Specific-Cause Mortality in the National Health and Nutrition Examination Survey, 2005 to 2008

Si-Yu Gui, Xin-Chen Wang, Jian-Chao Qiao, Si-Yu Lin, Qian-Qian Wang, Meng-Yue Zhang, Yue-Yang Xu, Zhi-Hao Huang, Li-Ming Tao, Cheng-Yang Hu*, Fang-Biao Tao*, Zheng-Xuan Jiang*, Dong-Wei Liu^*^

*** Correspondence:** Cheng-Yang Hu: ahmucy.hu@gmail.com

Fang-Biao Tao: taofangbiao@126.com

Zheng-Xuan Jiang: [jiangzhengxuan@ahmu.edu.cn](mailto:jiangzhengxuan@ahmu.edu.cn)


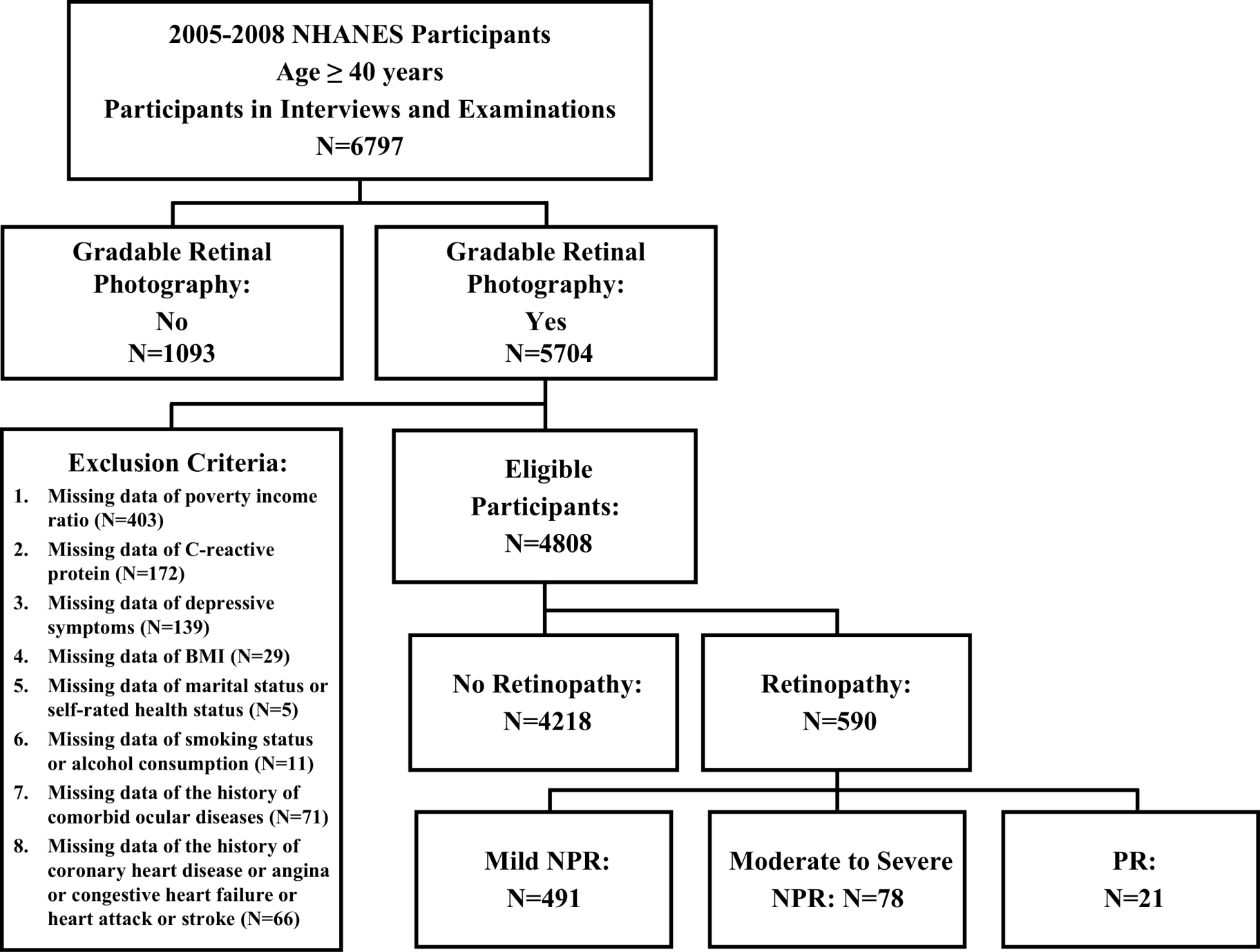


**eFigure.** Schematic Showing Inclusion Criteria for Study Participants. Schematic showing study participants included for the present analysis from the 2005-2008 National Health and Nutrition Examination Survey (NHANES). A total of 4808 participants were included.

# eTable 1. Demographic, Health Behavior, and General Health Characteristics of Participants by Retinopathy Status after PSM ^a^.

| **Characteristic** | **Study Participants after PSM** | | | |
| --- | --- | --- | --- | --- |
|  | **All**  **(n ^b^= 1180)**  **N ^c^= 9,269,922** | **No Retinopathy**  **(n = 590)**  **N = 4,697,061** | **Retinopathy**  **(n = 590)**  **N = 4,572,861** | ***P* value** |
| Age, No. (%), y |  |  |  | 0.98 |
| 40-49 | 202 (17.12) | 100 (24.02) | 102 (22.94) |  |
| 50-59 | 261 (22.12) | 132 (29.37) | 129 (30.18) |  |
| 60-69 | 367 (31.1) | 175 (23.00) | 192 (23.90) |  |
| 70-79 | 234 (19.83) | 123 (15.99) | 111 (15.20) |  |
| ≥80 | 116 (9.83) | 60 (7.62) | 56 (7.79) |  |
| Sex, No. (%) |  |  |  | 0.5 |
| Male | 700 (59.32) | 359 (55.95) | 341 (58.03) |  |
| Female | 480 (40.68) | 231 (44.05) | 249 (41.97) |  |
| Race/Ethnicity, No. (%) |  |  |  | 0.9 |
| Non-Hispanic White | 539 (45.68) | 278 (70.80) | 261 (70.93) |  |
| Non-Hispanic Black | 335 (28.39) | 164 (14.12) | 171 (14.85) |  |
| Mexican American | 205 (17.37) | 100 (6.88) | 105 (6.96) |  |
| Other | 101 (8.56) | 48 (8.20) | 53 (7.27) |  |
| Marital status, No. (%) |  |  |  | 0.3 |
| Unmarried or other | 424 (35.93) | 205 (28.63) | 219 (31.53) |  |
| Married or living with a partner | 756 (64.07) | 385 (71.37) | 371 (68.47) |  |
| Educational attainment, No. (%) |  |  |  | 0.85 |
| < High school | 433 (36.69) | 209 (23.86) | 224 (24.49) |  |
| ≥High school | 747 (63.31) | 381 (76.14) | 366 (75.51) |  |
| Poverty income ratio, No. (%) |  |  |  | 0.91 |
| Below poverty line (<1.00) | 191 (16.19) | 94 (9.54) | 97 (9.37) |  |
| At or above poverty line (≥1.00) | 989 (83.81) | 496 (90.46) | 493 (90.63) |  |
| Smoking status, No. (%) |  |  |  | 0.59 |
| No | 584 (49.49) | 298 (58.39) | 286 (57.01) |  |
| Yes | 596 (50.51) | 292 (41.61) | 304 (42.99) |  |
| Alcohol consumption, No. (%) |  |  |  | 0.69 |
| Never | 212 (17.97) | 111 (18.11) | 101 (16.68) |  |
| Former | 397 (33.64) | 197 (28.21) | 200 (29.94) |  |
| Mild | 324 (27.46) | 161 (29.41) | 163 (32.61) |  |
| Moderate | 111 (9.41) | 54 (10.27) | 57 (9.80) |  |
| Heavy | 136 (11.53) | 67 (14.00) | 69 (10.98) |  |
| BMI, No. (%) |  |  |  | 0.91 |
| 18.5-30.0 | 650 (55.08) | 321 (54.20) | 329 (55.21) |  |
| <18.5 | 10 (0.85) | 6 (0.48) | 4 (0.44) |  |
| ≥30.0 | 520 (44.07) | 263 (45.33) | 257 (44.34) |  |
| High C-reactive protein level, No. (%) |  |  |  | 0.44 |
| No | 1049 (88.9) | 531 (88.14) | 518 (89.84) |  |
| Yes | 131 (11.1) | 59 (11.86) | 72 (10.16) |  |
| Diabetes mellitus, No. (%) |  |  |  | 0.59 |
| No | 584 (49.49) | 298 (58.39) | 286 (57.01) |  |
| Yes | 596 (50.51) | 292 (41.61) | 304 (42.99) |  |
| Hypertension, No. (%) |  |  |  | 0.98 |
| No | 390 (33.05) | 211 (40.20) | 179 (40.28) |  |
| Yes | 790 (66.95) | 379 (59.80) | 411 (59.72) |  |
| Hyperlipidemia, No. (%) |  |  |  | 0.2 |
| No | 197 (16.69) | 97 (14.58) | 100 (17.91) |  |
| Yes | 983 (83.31) | 493 (85.42) | 490 (82.09) |  |
| Depressive symptoms, No. (%) |  |  |  | 0.69 |
| No | 1098 (93.05) | 549 (93.45) | 549 (93.95) |  |
| Yes | 82 (6.95) | 41 (6.55) | 41 (6.05) |  |
| Difficulty walking, No. (%) |  |  |  | 0.79 |
| No | 1017 (86.19) | 507 (87.93) | 510 (87.23) |  |
| Yes | 163 (13.81) | 83 (12.07) | 80 (12.77) |  |
| Health status, No. (%) |  |  |  | 0.74 |
| Poor to fair | 413 (35) | 188 (26.55) | 225 (27.48) |  |
| Good to excellent | 767 (65) | 402 (73.45) | 365 (72.52) |  |
| History of congestive heart failure, No. (%) |  |  |  | 0.92 |
| No | 1077 (91.27) | 543 (92.44) | 534 (92.25) |  |
| Yes | 103 (8.73) | 47 (7.56) | 56 (7.75) |  |
| History of coronary heart disease, No. (%) |  |  |  | 0.52 |
| No | 1062 (90) | 532 (89.05) | 530 (90.64) |  |
| Yes | 118 (10) | 58 (10.95) | 60 (9.36) |  |
| History of angina, No. (%) |  |  |  | 0.13 |
| No | 1108 (93.9) | 557 (95.65) | 551 (94.08) |  |
| Yes | 72 (6.1) | 33 (4.35) | 39 (5.92) |  |
| History of heart attack, No. (%) |  |  |  | 0.52 |
| No | 1077 (91.27) | 548 (93.52) | 529 (92.37) |  |
| Yes | 103 (8.73) | 42 (6.48) | 61 (7.63) |  |
| History of stroke, No. (%) |  |  |  | 0.62 |
| No | 1052 (89.15) | 524 (89.51) | 528 (90.75) |  |
| Yes | 128 (10.85) | 66 (10.49) | 62 (9.25) |  |
| History of cancer, No. (%) |  |  |  | 0.05 |
| No | 1031 (87.37) | 512 (84.35) | 519 (88.99) |  |
| Yes | 149 (12.63) | 78 (15.65) | 71 (11.01) |  |
| History of comorbid ocular diseases, No. (%) |  |  |  | 0.28 |
| No | 815 (69.07) | 413 (75.85) | 402 (72.51) |  |
| Yes | 365 (30.93) | 177 (24.15) | 188 (27.49) |  |

Abbreviations: NPR, Non-proliferative retinopathy; PR, proliferative retinopathy; PSM, Propensity score matching; BMI, body mass index (calculated as weight in kilograms divided by height in meters squared).

^a^ All proportions, means, and SEs are weighted estimates of the US population characteristics, taking into account the complex sampling design of the National Health and Nutrition Examination Survey (NHANES).

^b^ n represents the unweighted participant sample size.

^c^ N represents the representative population of non-institutionalized residents in the United States weighted according to the complex sampling design of NHANES.

# eTable 2. Demographic, Health-Related Behaviors and General Health Characteristics of Participants Included and Excluded in the Analyses ^a^.

| **Characteristic** | **No. of Included**  **Subjects (n = 4,808)** | **No. of Excluded**  **Subjects (n = 1,989)** | ***P* Value ^b^** |
| --- | --- | --- | --- |
| Age, No. (%), y |  |  | **< 0.0001** |
| 40-49 | 1303 (35.06) | 439 (28.86) |  |
| 50-59 | 1150 (30.28) | 372 (24.70) |  |
| 60-69 | 1210 (18.75) | 414 (16.98) |  |
| 70-79 | 789 (11.33) | 373 (14.43) |  |
| ≥80 | 356 ( 4.58) | 391 (15.02) |  |
| Sex, No. (%) |  |  | 0.06 |
| Male | 2417 (47.78) | 956 (44.98) |  |
| Female | 2391 (52.22) | 1033 (55.02) |  |
| Race/Ethnicity, No. (%) |  |  | **< 0.0001** |
| Non-Hispanic White | 2660 (78.54) | 886 (66.26) |  |
| Non-Hispanic Black | 956 ( 9.01) | 513 (15.23) |  |
| Mexican American | 731 (5.24) | 317 (6.43) |  |
| Other | 461 ( 7.21) | 273 (12.08) |  |
| Marital status, No. (%) |  |  | **< 0.001** |
| Unmarried or other | 1712 (30.39) | 864 (37.38) |  |
| Married or living with a partner | 3096 (69.61) | 1119 (62.62) |  |
| Educational attainment, No. (%) |  |  | **< 0.0001** |
| < High school | 1340 (17.09) | 801 (27.31) |  |
| ≥High school | 3468 (82.91) | 1177 (72.69) |  |
| Poverty income ratio, No. (%) |  |  | **< 0.0001** |
| Below poverty line (<1.00) | 724 ( 8.60) | 320 (15.24) |  |
| At or above poverty line (≥1.00) | 4084 (91.40) | 1140 (84.76) |  |
| Smoking status, No. (%) |  |  | **< 0.001** |
| No | 3731 (83.45) | 1405 (77.39) |  |
| Yes | 1077 (16.55) | 579 (22.61) |  |
| Alcohol consumption, No. (%) |  |  | **< 0.0001** |
| Never | 656 (10.95) | 284 (16.88) |  |
| Former | 1227 (20.91) | 442 (27.83) |  |
| Mild | 1688 (40.17) | 392 (32.07) |  |
| Moderate | 643 (15.18) | 133 (11.03) |  |
| Heavy | 594 (12.79) | 176 (12.19) |  |
| BMI, No. (%) |  |  | **0.02** |
| 18.5-30.0 | 2907 (61.68) | 1123 (63.28) |  |
| <18.5 | 63 (1.14) | 40 (2.48) |  |
| ≥30.0 | 1838 (37.18) | 667 (34.24) |  |
| High C-reactive protein level, No. (%) |  |  | 0.93 |
| No | 4237 (89.18) | 1425 (89.09) |  |
| Yes | 571 (10.82) | 196 (10.91) |  |
| Diabetes mellitus, No. (%) |  |  | **< 0.0001** |
| No | 3731 (83.45) | 1405 (77.26) |  |
| Yes | 1077 (16.55) | 579 (22.57) |  |
| Hypertension, No. (%) |  |  | **0.02** |
| No | 2192 (51.39) | 825 (46.55) |  |
| Yes | 2616 (48.61) | 1162 (53.45) |  |
| Hyperlipidemia, No. (%) |  |  | **< 0.0001** |
| No | 936 (19.47) | 618 (30.43) |  |
| Yes | 3872 (80.53) | 1369 (69.57) |  |
| Depressive symptoms, No. (%) |  |  | 0.08 |
| No | 4424 (93.12) | 1208 (91.35) |  |
| Yes | 384 (6.88) | 134 (8.65) |  |
| Difficulty walking, No. (%) |  |  | **< 0.0001** |
| No | 4350 (92.60) | 1545 (81.57) |  |
| Yes | 458 ( 7.40) | 444 (18.43) |  |
| Health status, No. (%) |  |  | **< 0.0001** |
| Poor to fair | 1217 (17.72) | 737 (30.30) |  |
| Good to excellent | 3591 (82.28) | 1247 (69.70) |  |
| History of congestive heart failure, No. (%) |  |  | **< 0.0001** |
| No | 4605 (96.98) | 1796 (93.65) |  |
| Yes | 203 (3.02) | 158 (6.35) |  |
| History of coronary heart disease, No. (%) |  |  | **0.02** |
| No | 4536 (95.25) | 1798 (93.49) |  |
| Yes | 272 (4.75) | 143 (6.51) |  |
| History of angina, No. (%) |  |  | **0.01** |
| No | 4622 (96.84) | 1846 (95.06) |  |
| Yes | 186 (3.16) | 111 (4.94) |  |
| History of heart attack, No. (%) |  |  | **< 0.0001** |
| No | 4522 (95.43) | 1780 (91.68) |  |
| Yes | 286 (4.57) | 195 (8.32) |  |
| History of stroke, No. (%) |  |  | **< 0.0001** |
| No | 4564 (96.10) | 1782 (92.21) |  |
| Yes | 244 (3.90) | 186 (7.79) |  |
| History of cancer, No. (%) |  |  | **0.01** |
| No | 4209 (88.09) | 1697 (84.98) |  |
| Yes | 599 (11.91) | 282 (15.02) |  |
| History of comorbid ocular diseases, No. (%) |  |  | **< 0.0001** |
| No | 3652 (80.95) | 578 (57.14) |  |
| Yes | 1156 (19.05) | 578 (42.86) |  |
| Mortality leading |  |  | **< 0.0001** |
| Cancer-Specific | 279 (4.13) | 149 (6.33) |  |
| CVD-Specific | 433 ( 6.48) | 285 (11.88) |  |
| DM-Specific | 40 (0.61) | 26 (0.97) |  |
| Other causes | 412 ( 6.17) | 303 (13.50) |  |
| No | 3644 (82.61) | 1226 (67.32) |  |
| Mortality status |  |  | **< 0.0001** |
| Assumed alive | 3644 (82.61) | 1224 (67.29) |  |
| Assumed deceased | 1164 (17.39) | 763 (32.71) |  |
| Retinopathy status |  |  |  |
| None | 4218 (90.49) | 776 (88.64) |  |
| Any Retinopathy | 590 ( 9.51) | 120 (11.36) | 0.11 |
| Mild NPR | 491 (8.31) | 82 (9.01) | **0.01** |
| Moderate to Severe NPR | 78 (1.00) | 28 (1.69) |  |
| PR | 21 (0.20) | 10 (0.66) |  |

Abbreviations: NPR, Non-proliferative retinopathy; PR, proliferative retinopathy; BMI, body mass index (calculated as weight in kilograms divided by height in meters squared).

^a^ Mortality was assessed through December 31, 2020. All proportions, means, and SEs are weighted estimates of the US population characteristics, considering the complex sampling design of the National Health and Nutrition Examination Survey (NHANES).

^b^ All *P* values were calculated using the unpaired t test for continuous variables and the design-adjusted Rao-Scott Pearson χ^2^ test for categorical variables. Comparisons were between each group with retinopathy and the group with no retinopathy and were unadjusted.

^*^ *P*<0.05; ^**^ *P*<0.01; ^***^ *P*<0.001; ^****^ *P*<0.0001.

# eTable 3. Cox Proportional Hazards Models for All-Cause Mortality and Fine and Gray Competing Risks Regression Models for Specific-Cause Mortality by Retinopathy Status after PSM ^a^.

| **Retinopathy Status** | **Mortality** | | | | |
| --- | --- | --- | --- | --- | --- |
|  | **HR (95% CI)** | | | | |
|  | **Due to all causes** | **Due to cancer** | **Due to CVD** | **Due to DM** | **Due to other causes** |
|  | **n = 400**  **N = 2,681,503** | **n = 84**  **N = 477,601** | **n = 154**  **N = 1,054,657** | **n = 27**  **N = 184,599** | **n = 135**  **N = 964,645** |
| **No Retinopathy**  n = 590  N = 4,697,061 | 1 [Reference]  No. (%) 413 (75.28)  N = 2,190,817 | 1 [Reference]  No. (%) 45 (5.26)  N = 247,198 | 1 [Reference]  No. (%) 59 (8.12)  N = 381,517 | 1 [Reference]  No. (%) 8 (1.24)  N = 583,288 | 1 [Reference]  No. (%) 65 (10.09)  N = 473,960 |
| **Retinopathy**  n = 590  N = 4,572,861 | 1.40 (1.01 to 1.93)^*^  No. (%) 367 (66.75)  N = 2,207,542 | 1.08 (0.57 to 2.05)  No. (%) 39 (5.04)  N = 230,403 | 1.91 (1.20 to 3.04)^**^  No. (%) 95 (14.72)  N = 673,139 | 2.45 (1.00 to 6.04)^*^  No. (%) 19 (2.76)  N = 1,262,706 | 1.17 (0.75 to 1.82)  No. (%) 70 (10.73)  N = 490,685 |
| *P* value | 0.04 | 0.81 | 0.01 | 0.05 | 0.49 |
| Abbreviations: CVD, cardiovascular disease; DM, Diabetes mellitus; PSM, Propensity score matching;HR, hazard ratio; CI, confidence interval.  ^a^ All proportions are weighted estimates of the US population characteristics, taking into account the complex sampling design of the National Health and Nutrition Examination Survey (NHANES).  ^b^ n represents the unweighted participant sample size.  ^c^ N represents the representative population of non-institutionalized residents in the United States weighted according to the complex sampling design of NHANES.  ^*^ *P*<0.05; ^**^ *P*<0.01; ^***^ *P*<0.001; ^****^ *P*<0.0001. | | | | | |

# eTable 4. Summary Description of Previous Studies on the Association Between Retinopathy and Mortality.

| **Study** | **Country** | **Age**  **range** | **Population** | **Median**  **follow-up (years)** | **HR (95% CI)** | **Statistical analysis** | **Retinopathy status** | **Adjustment for covariates** |
| --- | --- | --- | --- | --- | --- | --- | --- | --- |
| The Hoorn Study  2003 [33] | Netherlands | Not clear | 631  Nondiabetic and diabetic | 10.7 | All-cause:  Retinopathy vs no retinopathy: 1.4 (0.9-2.1) | Cox proportional hazards  analyses | Proliferative and nonproliferative retinopathy | Age, sex, diabetes and diabetes duration, BMI, prior cardiovascular disease, triglycerides, glycated hemoglobin, hypertension, smoking, homocysteine. |
|  |  |  |  |  | CVD-cause:  Retinopathy vs no retinopathy: 1.4 (0.7-2.8) |  |  |  |
| The Beaver Dam Eye Study  2007 [34] | USA | 43-84 | 4294 | 14 | All-cause:  Hemorrhages only vs no retinopathy: 0.95 (0.65-1.39)  Microaneurysms only vs no retinopathy: 1.02 (0.79-1.31)  Moderate retinopathy vs no retinopathy: **1.63 (1.01-2.64)**  Any retinopathy vs no retinopathy: 1.05 (0.86-1.28) | Cox proportional hazards  model | 1. no retinopathy, 2. presence of retinal hemorrhages only, 3. presence of retinal microaneurysms only, 4. presence of moderate or worse retinopathy. | Age, sex, pulse rate, diastolic blood pressure, smoking (pack-years), presence of proteinuria, history of cardiovascular disease, history of cancer, sedentary lifestyle, and use of diuretics. |
| The Ibaraki Prefectural Health Study  2011 [35] | Japan | 40-79 | 87890  With and  without hypertension | 14.1 | All-cause:  Men:  Grade 1 vs normal: **1.09 (1.04-1.15)**  Grade 2 vs normal: **1.17 (1.06-1.28)**  Women:  Grade 1 vs normal: 1.02 (0.96-1.08)  Grade 2 vs normal: **1.23 (1.11-1.35)** | Cox proportional hazards regression model | 1. normal: without retinopathy, 2. grade 1: mild narrowing or sclerosis of the retinal arterioles, 3. grade 2: moderate to marked sclerosis of the retinal arterioles, moderate narrowing of the retinal arterioles, or arteriosclerotic retinopathy or thrombosis of retinal veins. | Age, body mass index, systolic blood pressure, antihypertensive medication use (yes or no), serum total cholesterol  level, serum high-density lipoprotein cholesterol level, antidyslipidemic medication use (yes or no), blood glucose level (normal, prediabetes, and diabetes mellitus), antidiabetic medication use (yes or no), atrial fibrillation (yes or no), ST-T abnormality (yes or no), smoking status (never smoker, ex-smoker, currently smoking 20 cigarettes a day, currently smoking 20 cigarettes a day), and alcohol intake (never, sometimes, 44 g/d almost every day, and 44 g/d almost every day). |
|  |  |  |  |  | CVD-cause:  Men:  Grade 1 vs normal: **1.24 (1.12-1.38)**  Grade 2 vs normal: **1.23 (1.03-1.47)**  Women:  Grade 1 vs normal: **1.12 (1.01-1.24)**  Grade 2 vs normal: **1.44 (1.24-1.68)** |  |  |  |
| NHANES  2014 [15] | USA | ≥40 | 7640 | 14.5 | All-cause:  Retinopathy vs no retinopathy: 1.02 (0.75-1.38) | Cox proportional hazards analyses | With and without retinopathy. | Age, gender, race/ethnicity, education and annual family income, smoking status, hypertension, hemoglobin A1C, diabetes, cardiovascular disease, family history of coronary heart disease, body mass index, and total cholesterol. |
|  |  |  |  |  | CVD-cause:  Retinopathy vs no retinopathy: 0.96 (0.50-1.84) |  |  |  |
| AGES  2016 [19] | USA | 67-96 | 4966 | 8.6 | All-cause:  All Participants:  Retinopathy vs no retinopathy: 1.12 (0.97-1.30)  Men:  Retinopathy vs no retinopathy: **1.28 (1.04-1.57)**  Women:  Retinopathy vs no retinopathy: 0.98 (0.78-1.22) | Cox  proportional hazards regression model | 1. no retinopathy 2. mild non-proliferative retinopathy, 3. moderate-to-severe non-proliferative retinopathy or proliferative retinopathy. | Age, sex, smoking status, BMI, hypertension, diabetes, self-reported health status, cognitive status, walking disability, number of medications, and serum total cholesterol. For cardiovascular disease related mortality analyses, it further adjusted for self-reported history of angina or cardiovascular disease, microalbuminuria and chronic kidney disease, total brain tissue volume (corrected for intracranial volume), cerebral microbleeds, brain Infarcts, clinical stroke. |
|  |  |  |  |  | CVD-cause:  All Participants:  Retinopathy vs no retinopathy: 1.24 (0.91-1.69)  Men:  Retinopathy vs no retinopathy: **1.74 (1.14-2.66)**  Women:  Retinopathy vs no retinopathy: 0.95 (0.59-1.53) |  |  |  |
| NHANES  2018 [21] | USA | ≥40 | 4777  Without coronary artery disease, congestive heart failure, heart attack, or stroke at the baseline assessment | 4.6 | All-cause:  Mild vs no retinopathy: **1.81 (1.29-2.55)**  Moderate or severe vs no retinopathy: **4.14 (1.77-9.69)** | Cox proportional hazards model | 1. no retinopathy, 2. mild retinopathy, 3. moderate or severe retinopathy. | Age (years; continuous), sex, race/ethnicity (Mexican American, non-Hispanic white, non-Hispanic black, or other), self-reported smoking status (current, former, or never smoker), self-reported physical activity (MET-minutes/month; continuous), measured BMI (kg/m^2^; continuous), diabetes status (yes or no), hypertension (yes or no), and objectively measured visual acuity (normal vision, uncorrected refractive effort, or vision impairment). |
| NHANES  2021 [22] | USA | ≥40 | 5703 | 8.3 | All-cause:  Retinopathy vs no retinopathy: **1.41 (1.08-1.83)** | Cox proportional hazards  model | With and without retinopathy. | Age, gender, race, education level, marital status, income status, BMI, smoking status, drinking status, hypertension, diabetes mellitus, hypercholesterolaemia, C-reactive protein, self-rated health status, walking disability, self-reported history of CVD and CKD. |
| Abbreviations: NHANES, The National Health and Nutrition Examination Survey; AGES, The Age, Gene/Environment Susceptibility Reykjavik Study; BMI, body mass index; CVD, cardiovascular disease; CKD, chronic kidney disease. | | | | | | | | |
