# Supplementary material for: Association of retinopathy with risk of all-cause and specific-cause mortality in the National Health and Nutrition Examination Survey, 2005 to 2008
Source: Front Public Health. 2023 Aug 23;11:1200925. doi: 10.3389/fpubh.2023.1200925 (PMC10482412; doi:10.3389/fpubh.2023.1200925)

Supplementary Material B

Association of Retinopathy with Risk of All-Cause and Specific-Cause Mortality in the National Health and Nutrition Examination Survey, 2005 to 2008

Si-Yu Gui, Xin-Chen Wang, Jian-Chao Qiao, Si-Yu Lin, Qian-Qian Wang, Meng-Yue Zhang, Yue-Yang Xu, Zhi-Hao Huang, Li-Ming Tao, Cheng-Yang Hu*, Fang-Biao Tao*, Zheng-Xuan Jiang*, Dong-Wei Liu^*^

*** Correspondence:** Cheng-Yang Hu: ahmucy.hu@gmail.com

Fang-Biao Tao: taofangbiao@126.com

Zheng-Xuan Jiang: [jiangzhengxuan@ahmu.edu.cn](mailto:jiangzhengxuan@ahmu.edu.cn)

#
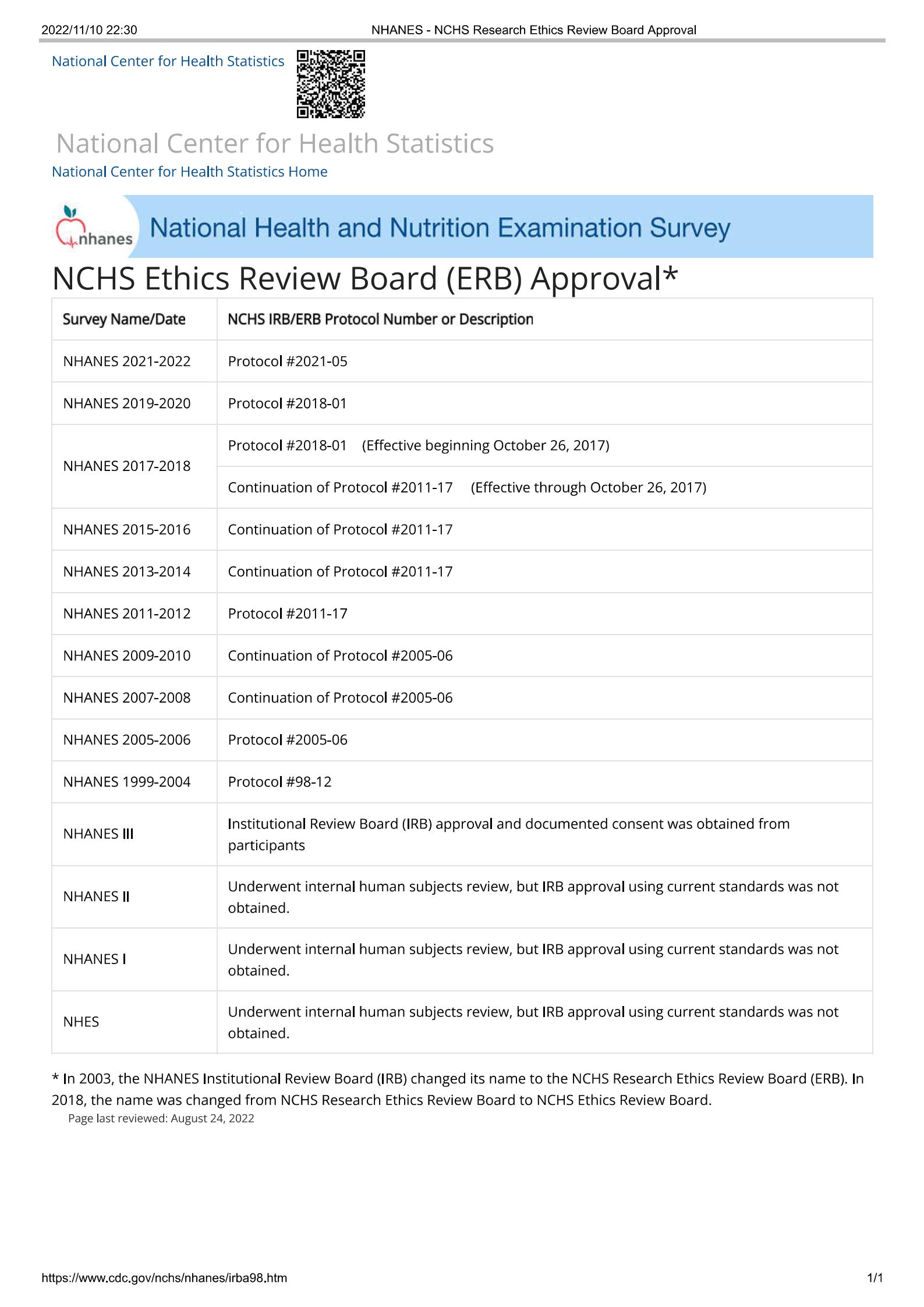

Supplement: Supplementary file 2 [file Table_2.DOCX]
